# Characteristics and transcriptional regulators of spontaneous epithelial–mesenchymal transition in genetically unperturbed patient-derived non-spindled breast carcinoma

**DOI:** 10.1186/s13058-024-01888-5

**Published:** 2024-09-10

**Authors:** Huang-Chun Lien, Hui-Chieh Yu, Wen-Hsuan Yu, Su-Fang Lin, Tom Wei-Wu Chen, I-Chun Chen, Li-Ping Hsiao, Ling-Chun Yeh, Yu-Chia Li, Chiao Lo, Yen-Shen Lu

**Affiliations:** 1 Department of Pathology, National Taiwan University Hospital, Taipei, Taiwan; 2 Graduate Institute of Pathology, National Taiwan University, Taipei, Taiwan; 3 Department of Oncology, National Taiwan University Hospital, No. 1, Changde St., Zhongzheng District, Taipei City, Taiwan; 4 Department of Medical Research, National Taiwan University Hospital, Taipei, Taiwan; 5 National Institute of Cancer Research, National Health Research Institutes, Miaoli County, Taiwan; 6 Department of Medical Oncology, National Taiwan University Cancer Center Hospital, Taipei, Taiwan; 7 Department of Surgery, National Taiwan University Hospital, Taipei, Taiwan

**Keywords:** Metaplastic breast carcinoma, Epithelial–mesenchymal transition, Mesenchymal–epithelial transition, Primary cell culture, Single-cell RNA sequencing

## Abstract

**Background:**

Although tumor cells undergoing epithelial–mesenchymal transition (EMT) typically exhibit spindle morphology in experimental models, such histomorphological evidence of EMT has predominantly been observed in rare primary spindle carcinomas. The characteristics and transcriptional regulators of spontaneous EMT in genetically unperturbed non-spindled carcinomas remain underexplored.

**Methods:**

We used primary culture combined with RNA sequencing (RNA-seq), single-cell RNA-seq (scRNA-seq), and in situ RNA-seq to explore the characteristics and transcription factors (TFs) associated with potential spontaneous EMT in non-spindled breast carcinoma.

**Results:**

Our primary culture revealed carcinoma cells expressing diverse epithelial–mesenchymal traits, consistent with epithelial–mesenchymal plasticity. Importantly, carcinoma cells undergoing spontaneous EMT did not necessarily exhibit spindle morphology, even when undergoing complete EMT. EMT was a favored process, whereas mesenchymal–epithelial transition appeared to be crucial for secondary tumor growth. Through scRNA-seq, we identified TFs that were sequentially and significantly upregulated as carcinoma cells progressed through the EMT process, which correlated with increasing *VIM* expression. Once upregulated, the TFs remained active throughout the EMT process. ZEB1 was a key initiator and sustainer of EMT, as indicated by its earliest significant upregulation in the EMT process, its exact correlation with *VIM* expression, and the reversal of EMT and downregulation of EMT-upregulated TFs upon *ZEB1* knockdown. The correlation between ZEB1 and vimentin expression in triple-negative breast cancer and metaplastic breast carcinoma tumor cohorts further highlighted its role. The immediate upregulation of ZEB2 following that of ZEB1, along with the observation that the knockdown of ZEB1 or ZEB2 downregulates both ZEB1 and ZEB2 concomitant with the reversal of EMT, suggests their functional cooperation in EMT. This finding, together with that of a lack of correlation of *SNAI1*, *SNAI2*, and *TWIST1* expression with the mesenchymal phenotype, indicated EMT-TFs have a context-dependent role in EMT. Upregulation of EMT-related gene signatures during EMT correlated with poor patient outcomes, highlighting the biological importance of the model. Elevated EMT gene signatures and increased *ZEB1* and *ZEB2* expression in vimentin-positive compared to vimentin-negative carcinoma cells within the corresponding primary tumor tissue confirmed ZEB1 and ZEB2 as intrinsic, instead of microenvironmentally-induced, EMT regulators, and vimentin as an in vivo indicator of EMT.

**Conclusions:**

Our findings provide insights into the characteristics and transcriptional regulators of spontaneous EMT in primary non-spindled carcinoma.

**Supplementary Information:**

The online version contains supplementary material available at 10.1186/s13058-024-01888-5.

## Introduction

Epithelial–mesenchymal transition (EMT), characterized by disruptions in cell adhesion and increased cell motility, is the process that enables cancer cells to migrate and invade other tissues [1, 2]. Although it is the common observation in laboratory experiments that tumor cells undergoing EMT often transition from structured, polarized epithelial units into single motile spindle cells, the presence of such a morphology remains a topic of debate in the field of pathology. This is because spindle morphology is rarely observed in primary carcinoma tissues across various organ systems, despite EMT being theoretically not an uncommon phenomenon in these tissues [3]. This rarity could be attributed to the transient and spatially variable nature of EMT, making it challenging to visualize in fixed tissue samples. To address this inconsistency, the concept of epithelial–mesenchymal plasticity (EMP) was introduced, proposing that cancer cells exhibit intermediate/hybrid epithelial–mesenchymal phenotypic states along the epithelial–mesenchymal spectrum [4]. However, the characteristics and underlying transcriptional regulators of spontaneous EMT in genetically unperturbed primary non-spindled carcinomas remain underexplored. Additionally, while certain markers, such as vimentin (Vim), may indicate EMT in tumor sections, their potential dynamic changes and functional significance have yet to be fully elucidated.

Changes in the expression of genes associated with EMT are predominantly governed by EMT transcription factors (EMT-TFs), such as Snail, Slug, ZEB1, ZEB2, and TWIST1, which play pivotal roles in driving the EMT process [4]. Evidence supporting these roles mainly comes from experimental studies manipulating key EMT regulators (e.g., knock-out/knock-in) and EMT lineage tracing models [5, 6]. Although these findings have substantially advanced the understanding of EMT, these approaches involve fixing cells at certain EMT stages and thus do not fully capture the physiological state in which cancer cells transiently and reversibly switch between various EMT states [5, 7]. Other studies on human tumors have investigated the effects of EMT-TFs [6]. Because tumor tissues contain a mixture of cancer cells progressing through EMT at different paces and shifting between epithelial and mesenchymal states, single-timepoint analyses during tumor progression may not fully reflect the effects of these TFs [5, 8]. Moreover, most studies have examined only a few EMT-TFs in the same context without exploring their potential relationships [6]. Although such studies have addressed fundamental questions regarding EMT-TFs, the potential upregulation of TFs and their relationships as cancer cells progress through spontaneous EMT in genetically unperturbed primary carcinoma remain underexplored.

Breast cancer is the most prevalent cancer among women and a major cause of mortality [9]. It is categorized into three major subtypes based on the presence or absence of molecular markers for estrogen or progesterone receptors and human epidermal growth factor receptor 2 (HER2): hormone receptor-positive/HER2-negative, HER2-positive, and triple-negative [10]. Metaplastic breast carcinoma (MpBC) is a highly aggressive subtype of breast cancer [11]. Typically, MpBC consists of triple-negative ductal carcinoma with diverse metaplastic components, such as squamous, spindle, and matrix-producing elements. These elements are believed to originate from ductal carcinoma through metaplastic processes [12–14]. Research has indicated that spindle metaplasia exhibits features characteristic of EMT [15–17]. By contrast, monophasic metaplastic squamous cell carcinoma (MMSCC), a variant of MpBC, features ductal carcinoma with varying degrees of squamous metaplasia without a spindle morphology. The presence of EMT in such non-spindled carcinoma remains unclear. In this study, we established a primary culture from a primary MMSCC sample to explore the potential existence, characteristics, and underlying regulators of spontaneous EMT in non-spindled breast carcinoma. Using this model, we validated and characterized the occurrence of spontaneous EMT, identifying sequentially upregulated TFs as the cancer cells underwent EMT. The findings of this study enhance the understanding of spontaneous EMT and the associated TFs in genetically unperturbed primary non-spindled carcinoma.

## Materials and methods

### Primary culture

This study was approved by the Institutional Review Board of National Taiwan University Hospital (201812045RIND). An MMSCC tumor sample preserved in CELLBANKER reagent was diced in digestion buffer and then placed in a dish with 3T3 feeder cells for cultivation in 2% IH medium. Supplementary Table S1 provides the details of the reagents used for digestion and culture.

### Tumor sample, immunohistochemistry, and immunofluorescence staining

Formalin-fixed, paraffin-embedded surgical specimens were obtained from the Department of Pathology at National Taiwan University Hospital. These specimens were retrieved from 20 patients with biphasic MpBC, which is characterized by both invasive carcinoma of no special type and spindle carcinomatous (SPS) components, and from 56 patients with triple-negative breast cancer (TNBC). Immunohistochemistry (IHC) was performed using an automated assay (Ventana, Oro Valley, AZ, USA), and immunofluorescence staining was performed following established procedures. Supplementary Table S1 lists the primary and secondary antibodies that were used.

### Western blotting and flow cytometry

For Western blotting, proteins extracted from cell lysates were separated on a sodium dodecyl sulfate polyacrylamide gel and then transferred to nitrocellulose membranes (Millipore, Bedford, MA, USA). Immunoreactive signals were detected using an enhanced chemiluminescence kit (WBKLS0500, Millipore). Flow cytometry was performed using FACS Calibur and CellQuestPro (BD Biosciences, San Jose, CA, USA). Supplementary Table S1 provides the details of the primary and secondary antibodies that were used.

### Quantitative reverse transcription polymerase chain reaction

Standard protocols were followed for the reverse transcription of total RNA to cDNA [18]. Quantitative reverse transcription polymerase chain reaction (qRT-PCR) was performed using the SYBR Green method on an ABI PRISM 7900 Sequence Detection System (Applied Biosystems, Foster City, CA, USA). The 2^−∆∆Ct^ method was used to calculate quantitative values. Normalization of the target gene measurements relative to GAPDH was performed for all samples. Supplementary Table S2 lists the primers used for qRT-PCR.

### Wound healing, migration, and invasion and anchorage-independent growth assays

The protocols followed for wound healing, migration, and invasion and anchorage-independent growth assays were described previously [19].

### Gene silencing

Lentiviral shRNA viral particles targeting *ZEB1* (TRCN0000017565), *ZEB2* (TRCN0000013530), and *CREB3L1* (TRCN0000015832) were obtained from the RNAi core laboratory at Academia Sinica (Taipei, Taiwan). An shRNA vector against lacZ (pLKO.1-shLacZ) was employed as a negative control. Cells were infected with the lentivirus for 24 h and then subjected to puromycin selection.

### Animal experiments

Twelve female NOG mice aged 14–18 weeks were divided into HE, HM, and control groups comprising five, five, and two mice, respectively. In the HE and HM groups, 1.5 × 10^6^ cancer cells were orthotopically injected into each mouse’s mammary fat pad. After 58 days, the mice were sacrificed, and the mammary fat pads were collected for analysis. All animal experimental procedures were approved by the Institutional Animal Care and Use Committee of National Taiwan University (20180361) and the National Health Research Institutes, Taiwan (NHRI-IACUC-106057-M1-A).

### 10x Genomics scRNA-seq data analysis

We used the Cell Ranger software pipeline (version 7.2.0, 10x Genomics) to demultiplex cellular barcodes and accurately map reads to genomes. For downstream analysis, we employed R packages, including scater (version 1.30.1), scDblFinder (version 1.16.0), and Seurat (version 5.0.1). These packages were used for per-cell quality control, doublet detection, data normalization, dimension reduction through principal component analysis (PCA), and uniform manifold approximation and projection for dimension reduction (UMAP) as well as for identifying cell clusters and biomarkers. Cellular-level quality control was performed to identify outliers on the basis of the median and median absolute deviation (MAD) values for each cell’s unique gene detection, library size, and mitochondrial gene percentages. We removed outliers with values exceeding 5 times the MAD for unique gene detection, 5 times the MAD for library size, and 3 times the MAD for mitochondrial gene percentages. We retained genes expressed in more than five cells for gene-level quality control. We used the SCTransform method in Seurat to normalize and variance-stabilize the UMI-count matrix. Subsequently, we selected the top 3000 genes exhibiting high variability for PCA. We used the first 13 principal components to perform UMAP and graph-based clustering, which resulted in identification of 11 cell clusters. Furthermore, cells were categorized into six clusters on the basis of the expression level of *VIM*. Subsequently, we used Seurat’s FindMarkers function to identify differentially expressed genes (DEGs) in each VIM cluster.

### Enrichment analysis

We obtained molecular signatures for 50 hallmark gene sets from the Human Molecular Signature Database and conducted gene set variation analysis (GSVA; version 1.50.0), using the R package for gene set enrichment analysis (GSEA). We used the gene-set-by-cell matrix of GSVA enrichment scores to calculate median normalized enrichment scores (NESs) to assess the gene set enrichment for each VIM cluster. Subsequently, the hallmark gene set was sorted on the basis of the rank correlation coefficient obtained through Spearman’s rank correlation analysis. The raw and processed RNA-seq data are available through the Gene Expression Omnibus (GEO; https://www.ncbi.nlm.nih.gov/geo/; number: GSE261037; secure token: kzarwssmftghbgt).

### Survival analysis

Transcriptome profiling and clinical data for human breast invasive carcinoma were obtained from the Genomic Data Commons data portal (Data Release 39.0, downloaded on January 15, 2024; https://portal.gdc.cancer.gov/); the TNBC cohort was used for analysis. In addition, clinical outcome endpoints, including disease-free interval (DFI) and overall survival (OS) data, [20] were obtained from https://tcga.xenahubs.net. Survival analysis was conducted using the Kaplan–Meier method and Cox proportional hazards models with the survival (version 3.5-7) R package. We re-grouped VIM expression levels into the following categories: VIM 0–2, VIM 2–3, VIM 3–4, VIM 4–5, and VIM 5–6. We performed Differential Expression Gene (DEG) analysis on the VIM 2–3, VIM 3–4, VIM 4–5, and VIM 5–6 groups, respectively, using the VIM 0–2 group as a reference, to identify the up-regulated DEGs. We found the gene signature for each group by taking the intersection of the up-regulated DEGs and the gene sets of EMT from the Hallmark gene sets in MSigDB. Next, we used each gene signature and TCGA RNA-seq data to calculate GSVA enrichment scores. We calculated the mean value based on these scores and divided the patient cohort into Low and High groups for survival analysis.

### RNA sequencing data analysis

Total RNA was extracted and sent for RNA sequencing (RNA-seq) to Tri-I Biotech Inc. (Taipei, Taiwan). Transcriptome libraries from the mRNA fractions were prepared using the MGIEasy RNA Library kit (SE50) and sequenced on a BGISEQ-500 machine. A gene matrix comprising 13,490 genes (in transcript per million) was analyzed for DEGs by using the R package limma (version 3.40.6) or GSEA (Broad Institute, MA, USA). The R package pheatmap (version 1.0.12) was used to cluster and visualize the relative expression levels of DEGs. The raw and processed RNA-seq data generated are available through the GEO (GSE183905; secure token: abkbimucpdojtqz).

### In situ RNA sequencing

The Cancer Transcriptome Atlas DNA probes (NanoString, Seattle, WA, USA) were used to simultaneously profile the RNA expression of over 1800 genes from distinct regions of interest (ROIs) with spatial resolution. Syto13 and morphology marker antibodies against pan-cytokeratin (PanCK) and Vim (Supplementary Table S1) were used, and slides were loaded onto the GeoMx Digital Spatial Profiler (DSP). The entire slide underwent imaging, and ROIs were selected. GeoMx DSP was used to identify segments exhibiting positive immunofluorescent signals for Vim or PanCK. After the areas of illumination (AOIs) were defined, the GeoMx DSP was used to expose them to 385-nm light, which released indexing oligonucleotides that were subsequently collected using a microcapillary. Sequencing libraries were generated through PCR that incorporated photoreleased indexing oligonucleotides, AOI-specific adapter sequences, and unique i5 and i7 sample indices. The PCR products were pooled, purified, and sequenced on an Illumina NextSeq (Illumina, San Diego, CA, USA).

### EMT scoring

The EMT score was calculated based on the 76-gene expression signature by Byers et al. [21]. Each cell’s score was calculated as a weighted sum of 76 gene expression levels. The scores were then centered by subtracting the mean across all cells, resulting in a grand mean of zero. Negative scores can be interpreted as representing the mesenchymal phenotype, while positive scores indicate the epithelial phenotype.

## Results

### Primary culture establishment and characterization

**Fig. 1:**
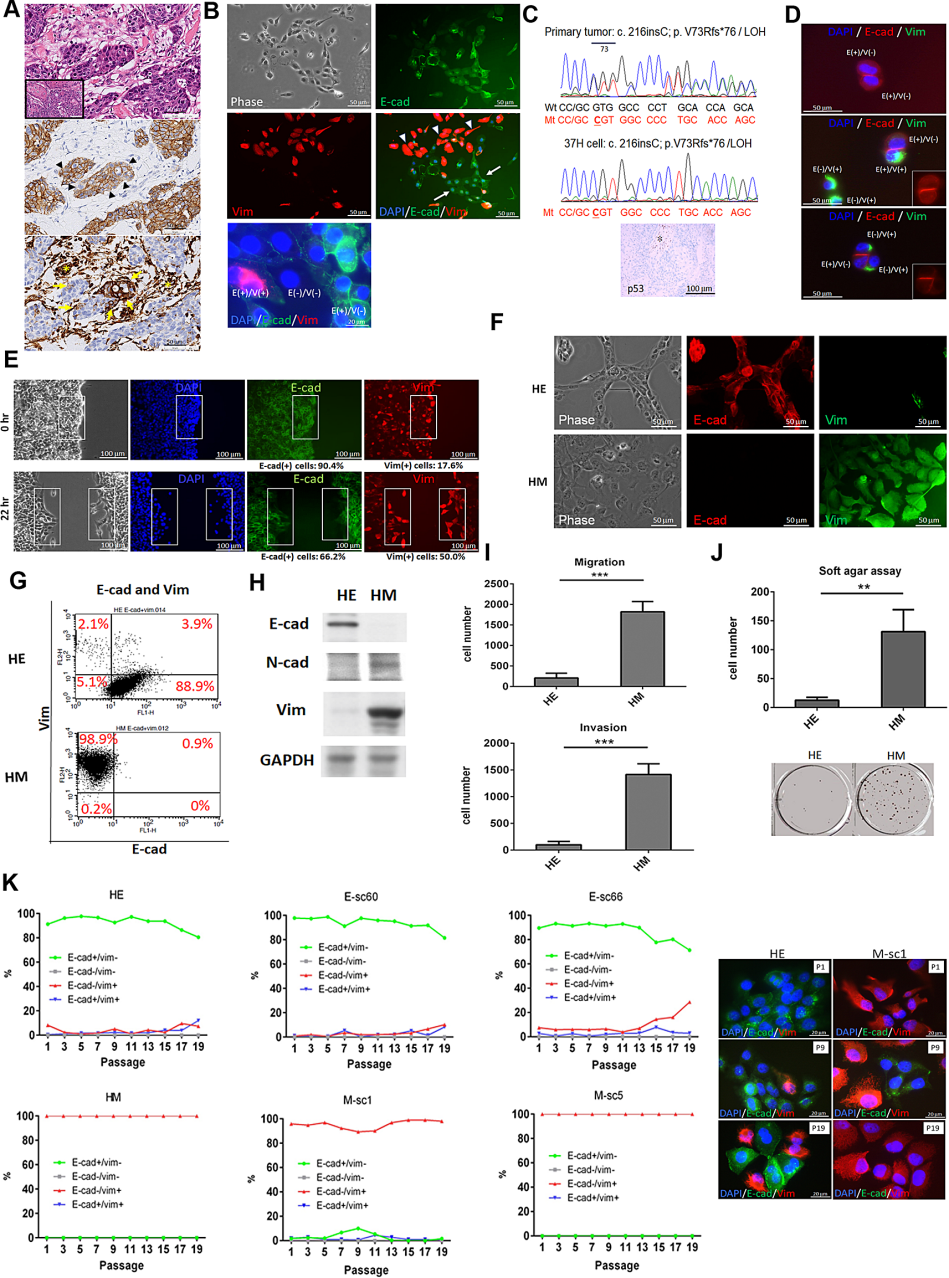
Establishment and phenotypic/functional characterization of 37 H primary culture cell lineages. **(A)** Histomorphology (hematoxylin–eosin staining) and immunohistochemistry (IHC) of the primary tumor tissue. The tumor was composed of conventional ductal carcinoma with focal squamous metaplasia (inset, upper panel). IHC staining for E-cad (middle panel) and Vim (lower panel) is presented. The carcinoma exhibits focal decreased staining for E-cad (arrowhead, middle panel) and increased staining for Vim (arrow, lower panel). The stroma serves as an internal control for Vim staining (star). **(B)** Phase-contrast and immunofluorescence staining of 37 H primary culture cells revealing a mix of compact polygonal E-cad^+^ Vim^−^ cells (arrow) and discohesive epithelioid E-cad^−^ Vim^+^ cells (arrowhead), with occasional E-cad^+^ Vim^+^ [E(+)/V(+)] and E-cad^−^ Vim^−^ [E(−)/V(−)] cells. **(C)** Sanger sequencing for *TP53* in the primary tumor tissue and 37 H cells. Loss of p53 expression in the tumor tissue was demonstrated through IHC (lower panel). Preserved p53 protein staining in the intervening stroma (star) served as the internal control. **(D)** Immunofluorescence staining for the E-cad/Vim (E/V) phenotypes of 37 H cells. E-cad staining is depicted in the inset image. **(E)** Wound healing assay results for 37 H cells, with percentages of E-cad^+^ cells and Vim^+^ cells before migration (0 h) and during migration (22 h after scratching). **F**–**H**. Phenotypes of HE and HM cells demonstrated through immunofluorescence staining (**F**), flow cytometry (**G**), and Western blotting (**H**). **I.** Migration and invasion ability of HE and HM cells, determined using the Transwell assay with and without Matrigel coating. **J.** Results of soft agar assay, performed to determine anchorage-independent growth ability (mean ± SD, **p < 0.01, ***p < 0.001, unpaired Student *t* test). **K.** Respective percentages of HE and HM cells and their progenies with E-cad^+^ Vim^−^, E-cad^−^ Vim^−^, E-cad^−^ Vim^+^, or E-cad^+^ Vim^+^ phenotype during culture for up to 19 passages. Tumor cells were seeded for immunofluorescence staining every two passages, and at least 300 tumor cells were evaluated from different views (magnification, 400×). Representative immunofluorescence images of HE cells and M-sc1 cells at passage 1 (P1), passage 9 (P9), and passage 19 (P19) are presented in the right panel

We performed primary culture of tumor tissue from a patient with MMSCC, identifying triple-negative ductal carcinoma with focal squamous metaplasia but no spindle histomorphology. The tumor cells predominantly exhibited intense E-cadherin (E-cad) staining, with some cells exhibiting decreased E-cad staining and increased Vim staining (Fig. 1A). In the corresponding 37 H primary culture, we observed clustered polygonal E-cad^+^ Vim^−^ cells, some single epithelioid E-cad^−^ Vim^+^ cells, and a few E-cad^+^ Vim^+^ cells and E-cad^−^ Vim^−^ cells (Fig. 1B). The absence of p63-positive tumor cells indicated that the 37 H cells had a ductal but not squamous origin of carcinoma (data not shown). Sequencing of both the 37 H cells and the primary tumor revealed an identical truncating *TP53* mutation (p.V73Rfs*76), confirming that they had a shared cellular origin (Fig. 1C). The presence of E-cad^−^ Vim^+^ or E-cad^+^ Vim^+^ cells adjacent to E-cad^+^ Vim^−^ cells supported the occurrence of spontaneous EMT (Fig. 1D). The wound healing assay revealed an enrichment of E-cad^−^ Vim^+^ cells at the migratory front, indicating a correlation between the E-cad^−^ Vim^+^ phenotype and enhanced migratory ability (Fig. 1E).

### 37 H cell lineage establishment and characterization

We established 37 H cell lineages enriched with E-cad^+^ Vim^−^ and E-cad^−^ Vim^+^ cells, which represented predominant epithelial and mesenchymal traits, respectively. Through dilution cloning, we isolated a clone primarily composed of E-cad^+^ Vim^−^ cells, hereafter referred to as HE cells (Fig. 1F). Because of the enhanced migratory nature of E-cad^−^ Vim^+^ cells, we subjected 37 H cells to a Transwell migration assay and collected the cells that moved through the membrane, obtaining a cell population predominantly consisting of E-cad^−^ Vim^+^ cells, hereafter referred to as HM cells. We characterized the immunophenotypes of these cells through immunofluorescence staining, flow cytometry, and Western blotting (Fig. 1F–H, Supplementary Fig. S1). Functional assays revealed that the HM cells exhibited higher migration, invasion, and anchorage-independent growth than did the HE cells (Fig. 1I, J). These findings confirmed that the HE and HM cells are representative of epithelial- and mesenchymal-predominant 37 H cells, respectively. Subsequently, we performed single-cell cloning of the HE and HM cells. The HE subclones included epithelial-predominant, mesenchymal-predominant, and mixed epithelial and mesenchymal cells, whereas the HM subclones primarily included mesenchymal-predominant cells (Supplementary Fig. S2 and S3). Additionally, mesenchymal-predominant M-sc1 and M-sc5 cells exhibited higher migration, invasion, and anchorage-independent growth than did the epithelial-predominant E-sc60 and E-sc66 cells (Supplementary Fig. S4 and S5). We explored the dynamic changes in the epithelial and mesenchymal phenotypes of the HE- and HM-type cells. All the three HE-type cells demonstrated spontaneous EMT during culture passages, as indicated by the gradual replacement of E-cad^+^ Vim^−^ cells with E-cad^+^ Vim^+^ and E-cad^−^ Vim^+^ cells (Fig. 1K). By contrast, the three HM-type cells consistently maintained a mesenchymal phenotype. Although the M-sc1 cells briefly underwent mesenchymal–epithelial transition (MET), they gradually reverted to the mesenchymal phenotype.

### Identification of transcriptional regulators involved in spontaneous EMT

**Fig. 2:**
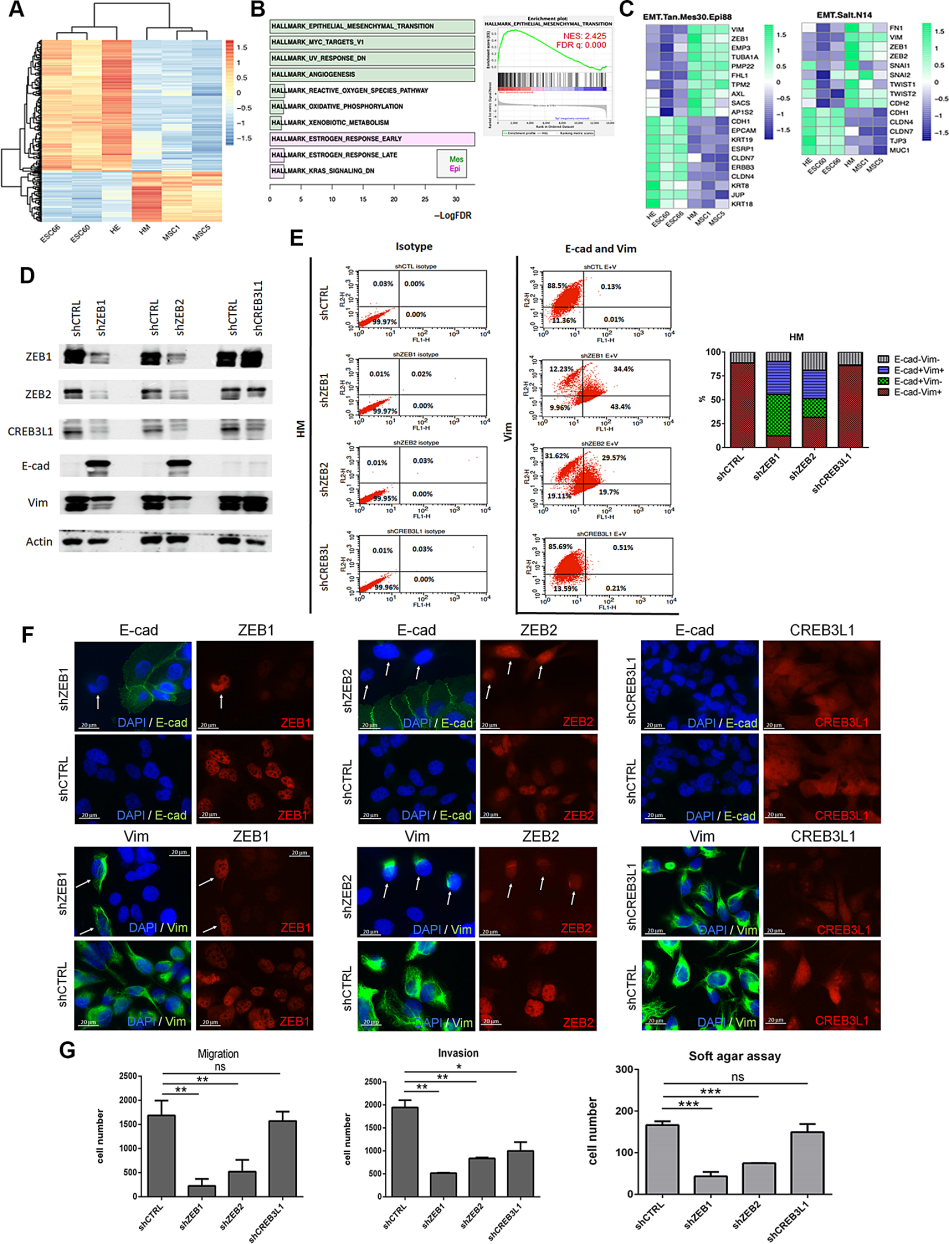
Identification and validation of spontaneous EMT regulators. **(A)** Heatmap showing DEG (limma adjusted *p* < 0.05) clustered by E (HE, E-sc60, and E-sc66) and M (HM, M-sc1, and M-sc5) groups. **(B)** Bar plot denoting statistical hallmark molecular pathways (FDR < 5%) enriched in the E and M groups, identified using GSEA. The EMT enrichment plot is presented on the right. NES, normalized enrichment score; FDR, false discovery rate. **(C)** Heatmaps depicting EMT signature genes identified by Tan et al. (PMID 25214461) and Salt et al. (PMID 24302555). **D**–**F.** Phenotypic changes in HM cells, assessed through Western blotting (**D**), flow cytometry (**E**), and immunofluorescence staining (**F**), following lentivirus-mediated knockdown of *ZEB1* (shZEB1), *ZEB2* (shZEB2), or *CREB3L1* (shCREB3L1) versus control (shCTRL). HM cells with incomplete knockdown of *ZEB1* or *ZEB2* (arrow) exhibited a mesenchymal phenotype, as evidenced by immunofluorescence staining results showing decreased E-cad and increased Vim (arrow). **G.** Transwell assay without (left panel) or with (middle panel) Matrigel coating and soft agar assay (right panel) performed to assess migration, invasion, and anchorage-independent growth, respectively, in HM cells with knockdown of *ZEB1*, *ZEB2*, or *CREB3L1* compared with that in a control knockdown (mean ± SD, *p < 0.05, **p < 0.01, ***p < 0.001, ns, not significant, unpaired Student *t* test)

Because HM cells are the mesenchymal counterparts of HE cells, the differentially enriched TFs in HM cells may serve as EMT regulators. To mitigate potential clonal heterogeneity, we included two subclones from each cell type in our RNA-seq analysis. The HE and HE-derived E-sc60 and E-sc66 cells formed the E group, whereas the HM and HM-derived M-sc1 and M-sc5 cells formed the M group. The clustering heatmap of DEGs revealed a strong genetic relatedness among the cells and subclones within the E and M groups (Fig. 2A). Subsequently, GSEA was conducted to identify differential hallmark molecular pathways enriched in the E and M groups. EMT, MYC, UV response, and angiogenesis were identified as the main mechanisms in the M group (Fig. 2B). MYC is involved in EMT function [22], and angiogenesis has been shown to link EMT-induced cancer stemness to tumor initiation [23]. Additionally, UV DNA damage has been shown to promote EMT-like changes [24]. The upregulation of these pathways in the M group is consistent with its functional correlation with EMT. Furthermore, GSEA performed using external gene sets associated with EMT [25, 26] indicated upregulation of the EMT signature in the M group (Fig. 2C). These results confirmed the suitability of our model for exploring EMT-TFs. To identify potential EMT-TFs in this model, we focused on TFs exhibiting expression levels in the M group that were higher than those in the E group. We combined the results of RNA-seq and the TRRUST database [27] and selected the top two TFs, namely HLX and CREB3L1, and the top two well-characterized EMT regulators, ZEB1 and ZEB2, as candidates for further validation. However, subsequent Western blotting analysis revealed considerably low HLX expression in the HM cells, leading us to exclude HLX from further analysis. To determine the involvement of the selected TFs in spontaneous EMT, we performed shRNA knockdown to silence the corresponding genes in the HM cells. We used pooled cells to reduce the effect of clone heterogeneity. Knockdown of ZEB1 or ZEB2 in the HM cells resulted in a transition from the mesenchymal (E-cad^−^ Vim^+^) to the epithelial (E-cad^+^ Vim^−^) phenotype (Fig. 2D–F, Supplementary Fig. S6), which was accompanied by reduced migration, invasion, and anchorage-independent growth (Fig. 2G). Notably, shZEB1 and shZEB2 downregulated ZEB1, ZEB2 and CREB3L1. By contrast, CREB3L1 knockdown in the HM cells did not result in a reversal to the epithelial phenotype (Fig. 2D–F) and had a moderate effect on invasion; no significant reduction in migration or soft agar colony formation was observed (Fig. 2G). These findings support a combined role for ZEB1 and ZEB2 in spontaneous EMT in this model.

### Association between expression of EMT-TFs and spontaneous EMT

**Fig. 3:**
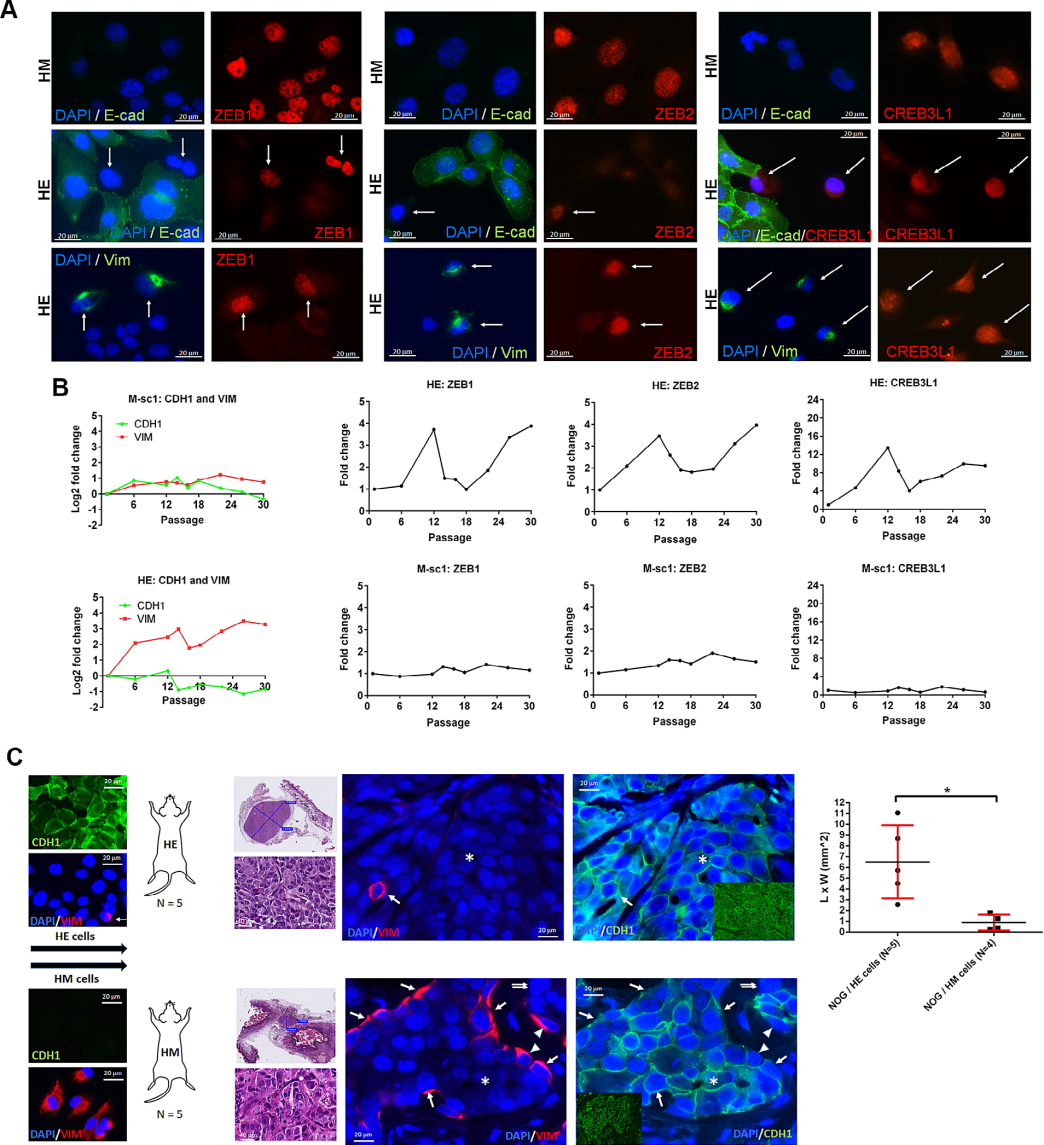
Expression of ZEB1, ZEB2, and CREB3L1 was correlated with the occurrence of spontaneous EMT. **(A)** Immunofluorescence staining revealing that ZEB1, ZEB2, and CREB3L1 were consistently expressed in HM cells (upper panel) but only observed in a few HE cells undergoing spontaneous EMT (arrow), as evidenced by a loss of E-cad expression (middle panel) or the presence of Vim (lower panel). **(B)** qRT-PCR for RNA expression abundance of *CDH1* and *VIM* and the three TFs: *ZEB1*, *ZEB2*, and *CREB3L1*. HE and HM cells were cultivated for 30 passages, and mRNA samples were collected and analyzed at passages 1, 6, 12, 14, 16, 18, 22, 26, and 30 through qRT-PCR. Quantitative values were determined on the basis of the threshold cycle number (Ct), and the fold-change in expression was calculated using the 2^−ΔΔCt^ method. Target gene measurements in all samples were normalized to the internal control gene *GAPDH*. The expression levels of corresponding genes on day 1 served as the baseline for result adjustments. **(C)** Spontaneous MET in HM cell xenografts. A schematic outline of the animal study workflow is shown (left panel). For each mouse, 1.5 × 10^6^ HE or HM cells mixed with Matrigel matrix were implanted into the mammary fat pads of 14–17-week-old female NOG mice (*n* = 5), and all tumors in the mammary fat pads were collected on day 58. Representative hematoxylin–eosin staining (magnification, 40× and 600×) and immunofluorescence staining for CDH1 and Vim with DAPI counterstaining for sample with HE or HM cells are presented (middle panel). Cancer cells exhibiting the CDH1^−^/Vim^+^, CDH1^+^/Vim^+^, CDH1^+^/Vim^−^, and CDH^−^/Vim^−^ phenotype are marked by an arrowhead, arrow, star, and double arrow, respectively. A lower power view for CDH1 staining is displayed in the inset image. The dot plot (right panel) illustrates the largest tumor size in each mouse implanted with HE or HM cells, measured as length (L) × width (W) (mean ± SD, *p < 0.05, unpaired Student *t* test)

We investigated the association between the expression of these TFs and the occurrence of spontaneous EMT. All three TFs were consistently expressed in mesenchymal HM cells. These TFs were also observed in HE cells undergoing spontaneous EMT, as indicated by expression of Vim or loss of E-cad expression (Fig. 3A). Subsequently, we investigated whether the TFs’ high expression levels were correlated with spontaneous EMT during culture passages. In line with the spontaneous EMT observed in the HE-type cells (Fig. 1K), qRT-PCR results indicated the occurrence of spontaneous EMT in the HE cells that was characterized by an increase in *VIM* expression and a concurrent decrease in *CDH1* expression (Fig. 3B). The trend of change in *ZEB1*, *ZEB2*, and *CREB3L1* expression aligned with spontaneous EMT. By contrast, the mesenchymal cells exhibited a more consistent mesenchymal phenotype. Overall, these findings indicate that ZEB1 and ZEB2 are EMT regulators, whereas CREB3L1 is a marker correlated with spontaneous EMT.

### Essential role of MET in the growth of tumor implants

We conducted a xenograft study to determine whether the tumor growth of HM cells exhibiting EMT phenotypes surpassed that of HE cells (Fig. 3C). Tumor formation was observed in all five HE cell xenografts but only in four out of five HM cell xenografts. Furthermore, lower tumor growth was observed in HM cell xenografts than in HE cell xenografts. In addition, in contrast to the predominant E-cad^−^ Vim^+^ phenotype of HM parental cells, the majority of the cells in the four tumor implants exhibited an E-cad^+^ Vim^−^ profile, indicating that EMT did not confer a growth advantage to the implants and that MET is crucial for secondary tumor outgrowth.

### Validation of the occurrence of spontaneous EMT and establishment of its correlation with both ZEB1 and ZEB2 through scRNA-seq analysis

**Fig. 4:**
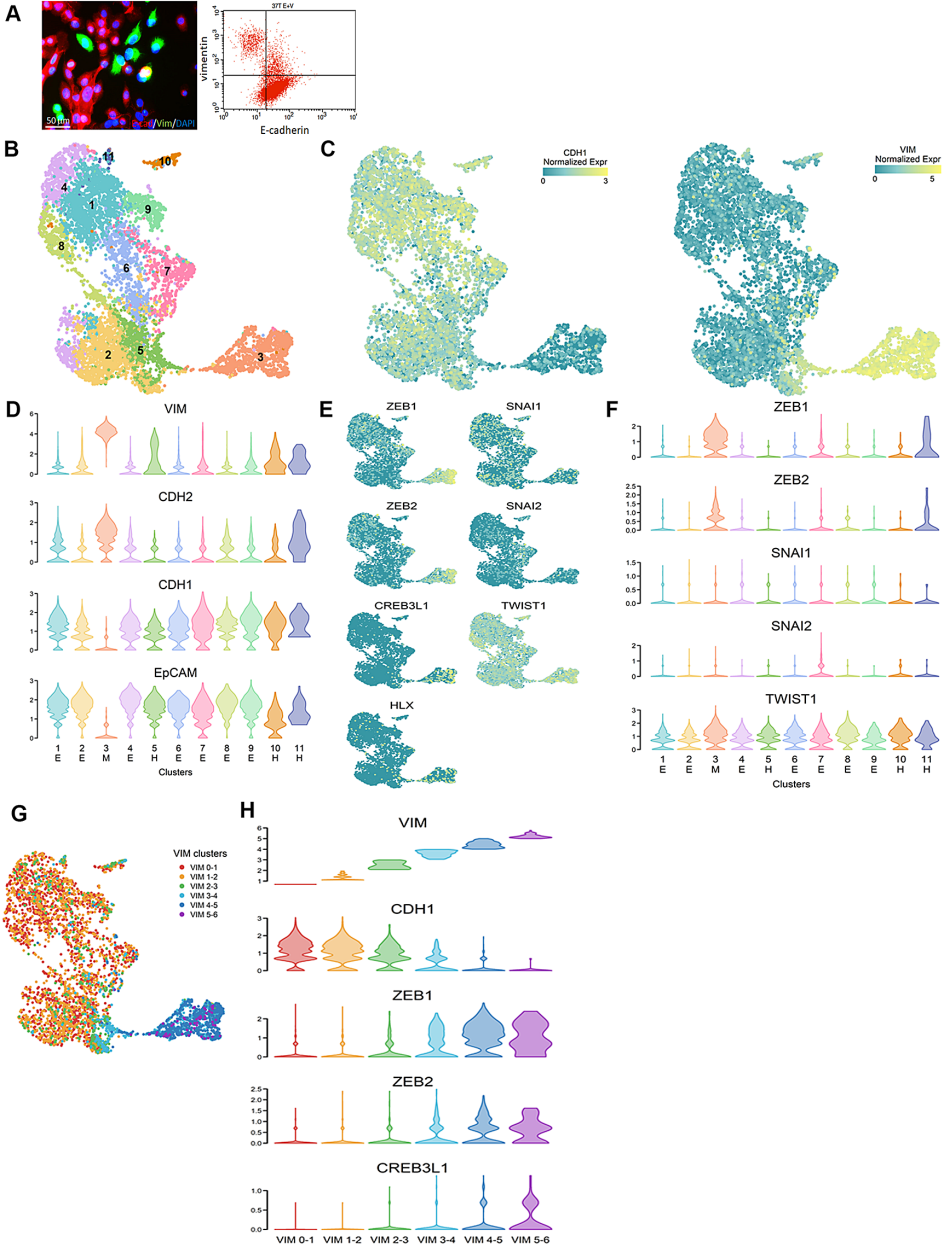
Overview of 7749 single cells derived from 37 H primary culture parental cells. **A.** Immunofluorescence staining (left) and flow cytometry (right) revealing expression of E-cad and Vim in 37 H cells. **B**,** C.** UMAP plots of the 7749 cells, color-coded by relevant cluster (**B**) or the expression of the marker gene *CDH1* (left) or *VIM* (right) (**C**). **D.** Violin plots illustrating the expression of the epithelial genes *VIM* and *CDH2* and the mesenchymal genes *CDH1* and *EpCAM* across the 11 identified clusters in the UMAP plot. These clusters are categorized as epithelial-predominant (E cluster: clusters 1, 2, 4, and 6–9), mesenchymal-predominant (M cluster: clusters 3), and hybrid (H cluster: clusters 5, 10, and 11) on the basis of the expression levels of these mesenchymal and epithelial markers. **E.** UMAP plots depicting the three TFs upregulated in HM cells, *ZEB1*, *ZEB2*, and *CREB3L1*, along with the three core EMT-TFs, *SNAI1*, *SNAI2*, and *TWIST1*. **F.** Violin plots displaying the expression levels of *ZEB1*, *ZEB2*, *SNAI1*, *SNAI2*, and *TWIST1* across the 11 clusters. **G** UMAP plots generated for cells stratified by *VIM* expression into six categories: VIM 0–1 (0 < log_2_*Vim* expression ≤ 1), VIM 1–2, VIM 2–3, VIM 3–4, VIM 4–5, and VIM 5–6. **H.** Violin plots illustrating expression of *ZEB1*, *ZEB2*, and *CREB3L1* and the epithelial marker *CDH1*, stratified by *VIM* expression

Although the HE and HM cells were enriched for the epithelial and mesenchymal phenotypes, respectively, both types of cells may include tumor cells progressing at various stages along the EMT/MET continuum, which could lead to heterogeneity in their gene expression. To investigate the characteristics and underlying regulators of spontaneous EMT and mitigate the potential effect of such heterogeneity, we conducted scRNA-seq on 37 H parental cells representing a spectrum of carcinoma cells advancing at different stages along the EMT course, as indicated by their phenotypes spanning the EMT spectrum (Fig. 4A).

As indicated by the expression of the mesenchymal markers *VIM* and *CDH2* and the epithelial markers *CDH1* and *EpCAM*, unsupervised UMAP revealed 11 clusters broadly corresponding to epithelial-predominant clusters (E cluster: clusters 1, 2, 4, and 6–9), a mesenchymal-predominant cluster (M cluster: clusters 3), and hybrid clusters (H cluster: clusters 5, 10, and 11; Fig. 4B–D). This finding confirms the presence of EMP, including cells from the CDH1^+^ VIM^−^ epithelial end to the CDH1^−^ VIM^+^ mesenchymal end. *ZEB1*, *ZEB2*, and *CREB3L1*, which were upregulated in mesenchymal-predominant HM cells (Supplementary Table S3), were enriched in the M cluster (Fig. 4E).

Whereas elevated expression of *ZEB1* and *ZEB2* in the M cluster and a lack of evident expression of *ZEB1* and *ZEB2* in the E cluster were noted, such correlations were not observed in the remaining core EMT-TFs *SNAI1*, *SNAI2*, and *TWIST1* (Fig. 4E, F) or in the additional well-characterized EMT-associated TFs [4], with the exception of *ID1* (Supplementary Fig. S7). We additionally stratified the cells on the basis of their levels of expression of the mesenchymal marker *VIM*, that is, from the lowest (VIM 0–1) to the highest expression (VIM 5–6; Fig. 4G and Supplementary Fig. S8). *ZEB1* and *ZEB2* expression were both respectively positively and negatively correlated with *VIM* and *CDH1* expression, whereas *CREB3L1* upregulation was observed only in the mesenchymal end (Fig. 4H). Consistently, the expression of the mesenchymal and epithelial markers was respectively positively and negatively correlated with both *ZEB1* and *ZEB2* expression (Supplementary Fig. S9). Together, these findings indicate that ZEB1 and ZEB2 are spontaneous EMT regulators.

### Identification of the TFs upregulated sequentially as cancer cells progress through the EMT course and confirmation of ZEB1’s pivotal role as a regulator of spontaneous EMT

**Fig. 5:**
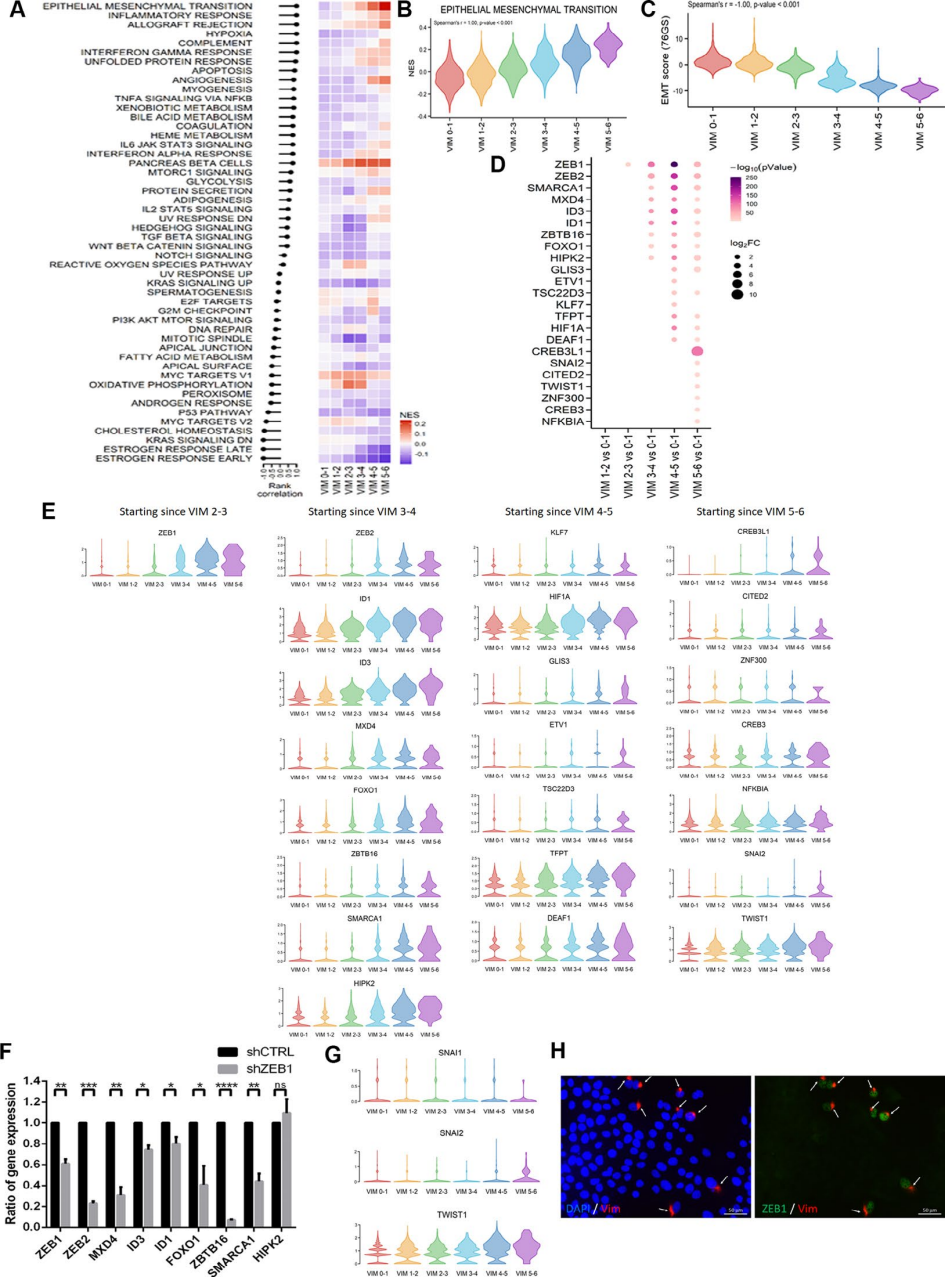
TFs that were differentially and significantly upregulated as cancer cells progressed through the EMT course, which correlated with increasing *VIM* expression. **(A)** Rank correlation of Hallmark signaling pathways. The *X* axis presents the expression status of *VIM* in cells, and the *Y* axis presents the NESs of molecular signatures. The correlation between *VIM* expression status and the NESs of molecular signatures was measured using Spearman’s rank correlation. **(B)** Violin plot illustrating hallmark EMT NESs of cells stratified by *VIM* expression abundance. **(C)** Violin plot illustrating EMT score (Byers LA et al.) of cells stratified by *VIM* expression abundance. **(D)** Bubble plot depicting differentially significantly upregulated TFs (*p* < 0.05, log_2_FC ≥ 1) between each VIM subgroup versus VIM 0–1 subgroup. **(E)** Violin plots illustrating expression levels of significantly upregulated TFs as cancer cells progress through the EMT course, which correlated with increasing *VIM* expression. **(F)** Bar plot illustrating the ratio of RNA level changes for early significantly upregulated TFs in mesenchymal-predominant HM cells following lentivirus-mediated knockdown of *ZEB1* (shZEB1) or control (shCTRL), assessed through qRT-PCR. *p < 0.05, **p < 0.01, ***p < 0.001, and ****p < 0.0001. ns, not significant. **(G)** Violin plots illustrating expression of *SNAI1*, *SNAI2*, and *TWIST1* stratified by *VIM* expression. **(H)** Immunofluorescence staining for ZEB1 and Vim in 37 H cells. Carcinoma cells expressing Vim are marked with arrows

We examined TFs that sequentially and significantly upregulated as cancer cells progressed through the EMT course, which correlated with the increasing expression of *VIM.* We discovered a highly significant correlation of high VIM expression levels with the NESs of the EMT pathway, as well as the EMT score [21], which provides strong support for the use of high VIM expression as an indicator of EMT status (Fig. 5A and C, Supplementary Table S4, and Supplementary Fig. S10). We identified *ZEB1* as the earliest significantly upregulated TF, with its upregulation beginning at the VIM 2–3 stage. Immediately after *ZEB1* was upregulated, *ZEB2*, *MXD4*, *ID1*, *ID3*, *SMARCA1*, *FOXO1*, *ZBTB16*, and *HIPK2* were significantly upregulated (VIM 3–4), whereas *CREB3L1*, *SNAI2*, *CITED2*, *TWIST1*, *ZNF300*, *CREB3*, and *NFKB1A* were significantly upregulated only at the final EMT stage (VIM 5–6; Fig. 5D, E, Supplementary Table S5 and Fig. S11). For the great majority of these TFs, after they were upregulated, they remained upregulated throughout the EMT course. The progressive increase in the expression of these early upregulated TFs (VIM 3–4) during EMT advancement (Fig. 5E), along with their heightened levels in the mesenchymal M cluster and their downregulation following ZEB1-knockdown-mediated EMT reversion (Fig. 5F), substantiates their role involving in acquiring the mesenchymal phenotype during the EMT process.

**Fig. 6:**
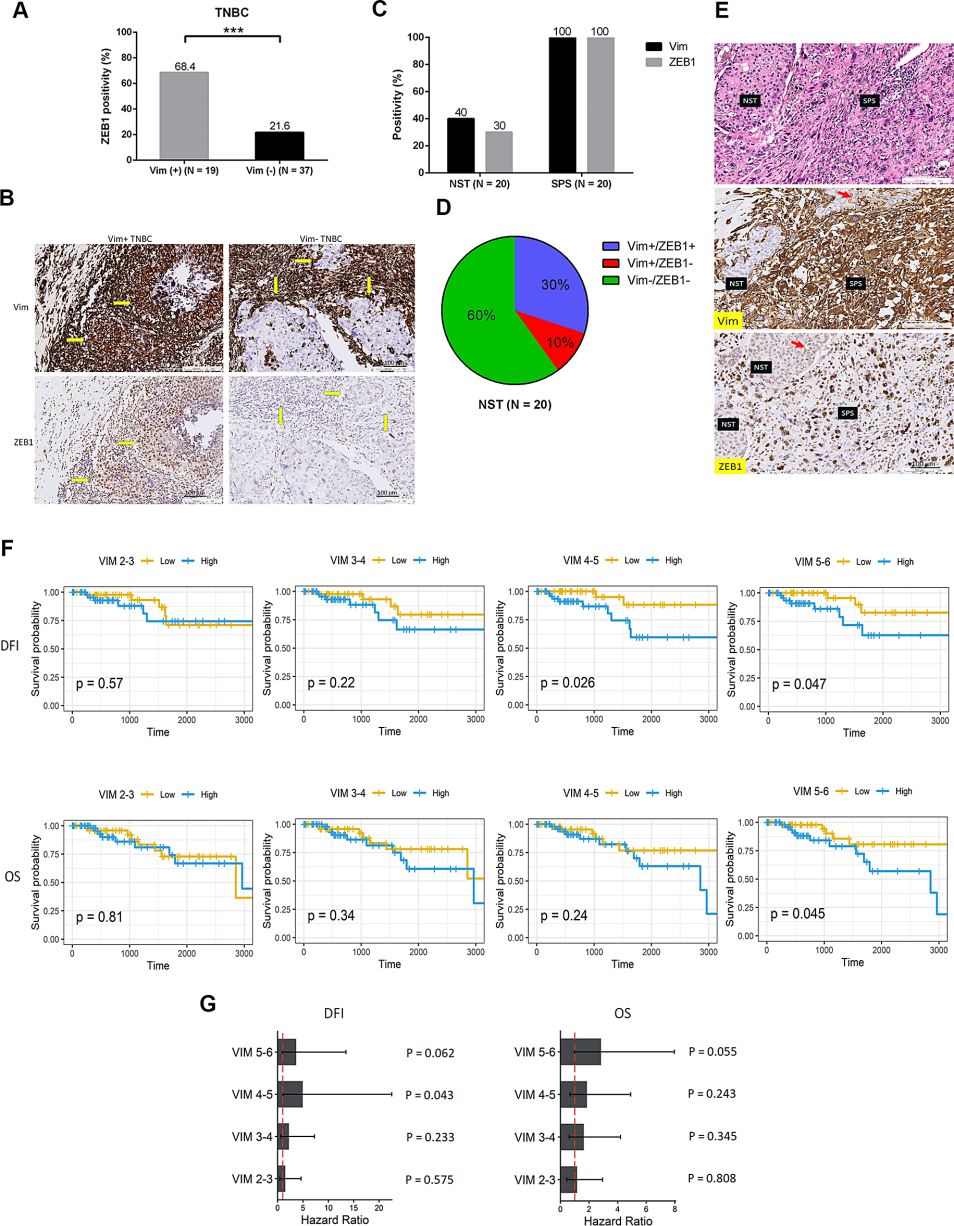
Correlation of ZEB1 with Vim expression and EMT in the TNBC and MpBC cohorts, and prognostic impact of upregulated gene signatures as cancer cells progress through the EMT process. **A**,** B.** Bar plot (**A**) illustrating the frequency of ZEB1 expression in 56 TNBC cases with or without Vim expression, accompanied by representative IHC (**B**) depicting Vim and ZEB1 expression. Arrows indicate carcinoma regions. **C.** Bar plot illustrating the positivity ratio of Vim and ZEB1 staining in the conventional ductal carcinoma (NST) components and spindle carcinomatous (SPS) components across 20 cases of MpBC with paired NST and SPS components. **D.** Pie plot illustrating the paired status of Vim and ZEB1 staining in the NST components in the 20 cases of MpBC. **E.** Histomorphology (hematoxylin–eosin staining; upper) and IHC staining for Vim (middle) and ZEB1 (lower) in paired NST and SPS components in a representative case of MpBC. The few ZEB1-stained cells are marked by an arrow (Magnification, 200×). **F**,** G.** Kaplan–Meier plots (**F**) and Cox proportional hazard models (**G**) used to compare disease-free intervals and overall survival among TCGA TNBC cases, stratified by high and low expression levels of differentially overexpressed EMT-related genes at various stages of VIM (VIM 2–3, VIM 3–4, VIM 4–5, and VIM 5–6) compared with those at VIM 0–2 stages. Samples were assessed through gene set variation analysis based on the expression values of differentially expressed genes and then categorized into high-expression and low-expression groups by using the mean value. The log-rank test was used to calculate *p* values

Unlike *ZEB1* and *ZEB2*, the core EMT regulators *SNAI2* and *TWIST1* were significantly upregulated only at the final stage of EMT (VIM 5–6), whereas *SNAI1* consistently maintained minimal expression throughout the entire EMT course (Fig. 5G and Supplementary Fig. S12, 13). The precise correlation between *ZEB1* and *VIM* expression during EMT progression (Fig. 5E) was further confirmed through immunofluorescence staining (Fig. 5H). Because the primary tumor exhibited triple negativity, we investigated the effect of ZEB1 on the upregulation of Vim in a TNBC cohort and identified a strong correlation between ZEB1 and Vim expression (Fig. 6A, B). We subsequently examined the involvement of ZEB1 expression in EMT acquisition in a cohort of MpBC with paired ductal carcinomatous and spindle metaplastic components, with spindle metaplastic components being considered the EMT counterpart of paired ductal carcinomatous components [15, 16, 28]. Our observations revealed coexpression of Vim and ZEB1 in all spindle metaplastic components but a significantly lower expression ratio of both markers in the paired carcinomatous components (Fig. 6C–E). The expression of ZEB2 was also enriched in spindle metaplastic components (Supplementary Fig. S14). In addition, we observed consistent expression of Vim and ZEB1 in the carcinomatous components. These findings confirm the pivotal role of ZEB1 as an EMT regulator in breast cancer.

### Unfavorable prognostic implications associated with gene signatures elevated during the EMT process

We investigated the prognostic implications of gene signatures that are upregulated during the EMT process, particularly those overlapping with genes in the hallmark EMT pathway. We determined that gene signatures overexpressed in cancer cells progressing through the EMT stages (VIM 2–6) compared with those in the VIM 0–2 stages demonstrated a trend toward an unfavorable prognostic effect on survival, DFI, and OS. This trend was particularly significant in the middle (VIM 4–5) and late (VIM 5–6) EMT stages (Fig. 6F, G). The results highlight the unfavorable prognostic significance of gene signatures being elevated during the EMT process, validating the biological relevance of this model.

### Elevated EMT gene signatures, along with increased expression of ZEB1, ZEB2, and CREB3L1, in Vim^+^ compared with Vim^−^ carcinoma cells within the corresponding primary tumor tissue

**Fig. 7:**
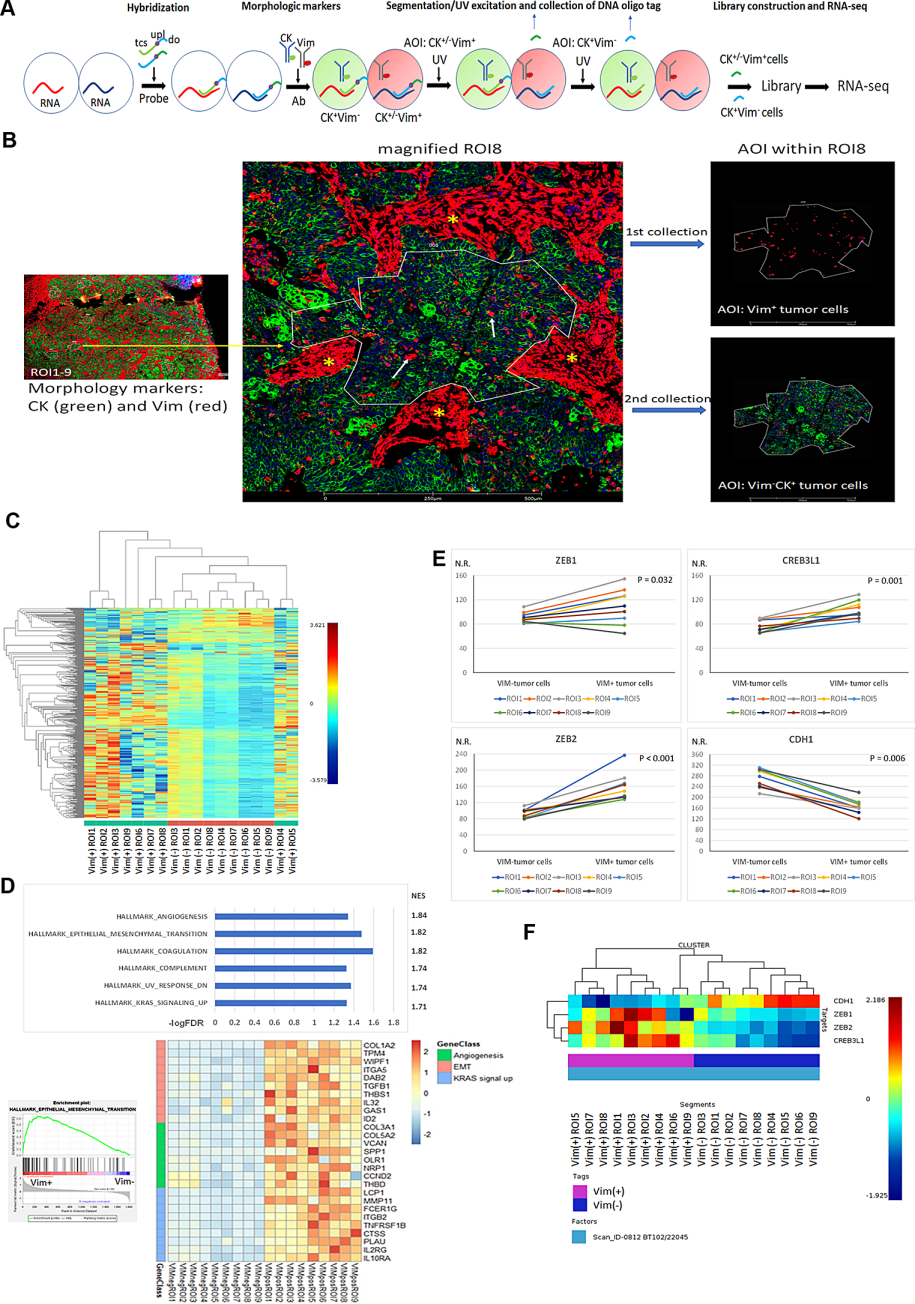
In situ validation of spontaneous EMT and EMT regulators/markers derived from the primary culture model. **(A)** Schematic of in situ RNA-seq (digital spatial profiling) of CK^+/−^ Vim^+^ versus CK^+^ Vim^−^ tumor cells from the primary tumor sample, determined using GeoMax RNA assays. tcs, target complementary DNA sequence; upl, UV photocleavable linker; do, DNA oligo tag; Ab, antibody. **(B)** Representative images of ROI and AOI segmentation in the primary tumor section. In total, nine ROIs (ROI1–9) were selected (white line) by using the morphology markers CK (green) to highlight carcinoma nests and Vim (red) to identify carcinoma cells expressing Vim (left panel). A magnified image of ROI8 is presented (middle panel), with Vim^+^ carcinoma cells within the ROI8 marked by an arrow. The intervening stroma components, which were Vim^+^ (star), were avoided when ROIs were selected. In ROI8, the AOI segmentation of Vim^+^ carcinoma cells (right upper panel) was UV illuminated; the DNA oligo tag was collected first, and the AOIs for Vim^−^ carcinoma cells were collected sequentially (right lower panel). **(C)** Unsupervised hierarchical clustering heatmap presenting clustering of expressed genes from paired Vim^+^ versus Vim^−^ carcinoma cell AOIs of the nine ROIs. **(D)** Bar plot depicting statistical hallmark molecular pathways (FDR < 5%) enriched in Vim^+^ carcinoma cells, determined by using GSEA (upper panel), as well as visualization of the EMT enrichment plot of EMT (left lower panel) and heatmap of leading genes associated with Vim^+^ group hallmark molecular processes (right lower panel). **E** Differences in expression of *ZEB1*, *ZEB2*, *CREB3L1*, and *CDH1* between paired Vim^+^ and Vim^−^ tumor cells from the nine ROIs are plotted, with the paired *t* test used for testing these differences. N.R., normalized reads. **F.** Heatmap of expression of *ZEB1*, *ZEB2*, *CREB3L1*, and *CDH1* in the nine ROIs

Finally, we validated our findings in the corresponding primary tumor tissue. Using DSP for RNA-seq [29], we compared the RNA expression in paired Vim^+^ versus Vim^−^ carcinoma cells collected from nine ROIs in the tumor tissue section (Fig. 7A, B). An unsupervised clustering heatmap revealed a clear distinction in gene expression between Vim^+^ and Vim^−^ carcinoma cells (Fig. 7C). Subsequently, GSEA revealed that the EMT hallmark played a significant role in distinguishing Vim^+^ from Vim^−^ carcinoma cells (Fig. 7D). Further analysis of differences in the expression of *ZEB1*, *ZEB2*, and *CREB3L1*, which are included in the GeoMx Cancer Transcriptome Atlas Panel gene list, revealed significant enrichment in Vim^+^ carcinoma cells relative to that in Vim^−^ carcinoma cells from the nine ROIs (Fig. 7E). For comparison, the expression of *SNAI2* and *TWIST1* is significantly higher in Vim^+^ tumor cells compared to Vim^−^ tumor cells, whereas SNAI1 expression shows no significant difference between Vim^+^ and Vim^−^ tumor cells (Supplementary Fig. S15). Moreover, a heatmap depicting *ZEB1*, *ZEB2*, *CREB3L1*, and *CDH1* across the nine ROIs revealed clear segregation between Vim^+^ and Vim^−^ carcinoma cells (Fig. 7F). These in vivo findings corroborate the data obtained from the primary culture model.

## Discussion

The findings of this study reveal that genetically unperturbed non-spindled carcinoma includes a spectrum of carcinoma cells exhibiting phenotypes ranging from the E-cad^+^ Vim^−^ epithelial end to the E-cad^−^ Vim^+^ mesenchymal end. This result supports the concept of EMP, where carcinoma cells differentiate along the epithelial–mesenchymal spectrum in primary tumors. EMT is characterized by a loss of proteins associated with the polarized epithelial phenotype and by synthesis of proteins associated with the mesenchymal morphology and involves a transition from polarized epithelial units to individual motile spindle cells [30]. Typically, the loss of E-cad and a switch in the usage of the intermediate filament from cytokeratin to Vim is observed in complete EMT [4]. Our study revealed that the majority of the cells in the M clusters, identified through scRNA-seq, exhibited a complete loss of *CDH1* and *EpCAM* accompanied by a significant increase in *VIM* and *CDH2* expression. These cells demonstrated enhanced migration, invasion, and anchorage-independent tumorigenicity, indicating that E-cad^−^ Vim^+^ cells represent the complete EMT counterpart of E-cad^+^ Vim^−^ cells. However, the HM cells maintained a single epithelioid morphology throughout the culture, deviating from the typical spindle morphology observed in the mesenchymal end of the EMT process in many experimental systems [4]. The current study’s discovery of an absence of spindle Vim^+^ tumor cells in xenografts and primary tumor sections challenges previous reports of an association of spindle morphology with the mesenchymal end of EMT. Our observation of high ZEB1 expression in the HM cells aligns with previous findings of elevated ZEB1 expression in cancer cells lacking morphological indicators of complete EMT [31, 32]. This finding demonstrates that EMT-TFs may be active, even in cells lacking overt spindle morphology [6]. Our results offer a plausible explanation for the discrepancy between current histopathological assessments and experimental systems, suggesting complete EMT in tumors does not always manifest as spindle morphology and that such morphology may be limited to rare tumor subtypes.

Although EMT and MET are widely recognized as crucial processes in tumor advancement [33], limited evidence has been provided of the dynamic changes in these processes in genetically unperturbed primary tumors. In this study, our primary culture system enabled sequential tracking of phenotypic changes in cancer cells; such a task is difficult to complete through static analyses of pathological tissue sections or tumor samples collected at specific points in tumor development [5]. Our findings indicate that all epithelial-predominant HE cells and their progenies exhibited spontaneous EMT during culture passages, suggesting that spontaneous EMT is a common event. By contrast, the consistent E-cad^−^ Vim^+^ phenotype observed in the HM cells and their progenies throughout culture passages implies that spontaneous MET is a less common event. This observation is corroborated by scRNA-seq data revealing the frequent presence of cancer cells expressing VIM in the HE cells or E cluster. Our findings align with those reported by Sarrio et al., who indicated that EpCAM^+^ mammary epithelial cells can spontaneously transition to mesenchymal EpCAM^−^ cells, whereas the reverse transition occurs less frequently [34]. Furthermore, the larger tumor size and the lack of evident EMT in the HE cell xenografts, along with the consistent presence of MET in the HM cell xenografts, suggest a distinct role of EMT and MET in tumor progression. While EMT is associated with increased invasiveness during tumor progression, MET appears to be essential for secondary tumor outgrowth. These results are consistent with previous observations of a reversion from a mesenchymal to an epithelial state during metastatic outgrowth in a mouse tumor model, even in the absence of experimental modifications to EMT inducers [7]. Together, these findings suggest that selective pressures during secondary tumor outgrowth enrich the epithelial sub-population, although the mechanism remains to be elucidated.

Through scRNA-seq, we comprehensively identified TFs that exhibited sequential and significant upregulation as cancer cells progressed through EMT. Our study identified *ZEB1* as the earliest TF to exhibit significant upregulation, with its upregulation coinciding with *VIM* expression. Notably, for the majority of these TFs, after they were activated, consistent upregulation was maintained throughout the entire EMT process. This finding indicates that EMT-upregulated TFs, once initiated, remain active in upregulating downstream TFs, which amplifies the cascade that underlies the complex event of EMT, instead of being sequentially activated at distinct EMT stages. The earliest upregulation of *ZEB1* during the initiation of the EMT process, the precise correlation between *ZEB1* and *VIM* expression identified through scRNA-seq and immunofluorescence staining, and the reversal of EMT upon *ZEB1* knockdown collectively confirm the pivotal role of ZEB1 in initiating and sustaining the EMT phenotype in breast cancer. Although our results are based on a single tumor sample, the significant correlation between Vim and ZEB1 expression in the TNBC cohort and the consistent ZEB1 expression in all spindle metaplastic components of MpBC, which is pathologically associated with EMT, substantiate the biological importance of ZEB1 in acquiring Vim expression and the EMT phenotype in breast cancer. This finding provides compelling in vivo evidence supporting ZEB1 as a core EMT-TF [4, 6], aligning with previous research highlighting enriched EMT features in TNBC cancer cells expressing Vim [35, 36] and linking EMT features with ZEB1 expression in tumor budding [31]. The current study findings have potential clinical implications: ZEB1-expressing cancer cells demonstrate high resistance to therapy but sensitivity to ferroptosis-inducing drugs [6, 37]. Moreover, aggressive tumors with an EMT phenotype often exhibit high expression of PD-L1 indirectly induced by ZEB1, resulting in immunosuppression in breast cancer [6, 38]. Combining ferroptosis-inducing drugs with immune checkpoint inhibitors may offer therapeutic benefits in carcinomas exhibiting an EMT phenotype.

In this study, we classified *ZEB2*, *MXD4*, *ID1*, *ID3*, *SMARCA1*, *FOXO1*, *ZBTB16*, and *HIPK2* as early upregulated TFs in the EMT process because of their upregulation (VIM 3–4) immediately following *ZEB1* upregulation (VIM 2–3). Thus, these TFs were considered more likely than late upregulated TFs to play a causal role in EMT. ZEB2 is well-recognized as a core EMT-TF [4, 6]. Although experimental evidence indicates potential context-dependent interactions among these core EMT-TFs [6, 39–41], such interactions in genetically unperturbed primary cancers remain underexplored. In this study, we identified and validated both ZEB1 and ZEB2 as spontaneous EMT regulators on the basis of their correlations with *VIM* expression, upregulation of *ZEB2* occurring promptly after upregulation of *ZEB1* in the EMT process, and the observed downregulation of both ZEB1 and ZEB2 concomitant with the reversal of EMT upon their knockdown. Our findings provide evidence for the combined role of ZEB1 and ZEB2 in the acquisition of the EMT phenotype in primary breast cancer.

The members of the ID gene family heterodimerize with basic helix-loop-helix (bHLH) TFs, which inhibits their DNA binding and affects development [42, 43]. Among these members, ID1 has been implicated in EMT in breast cancer [44]; the involvement of ID3 in EMT remains unclear. MXD4, part of the MAD gene family encoding bHLH TFs that heterodimerize with MAX protein, forms a transcriptional repression complex [45]. Although the role of MXD4 in EMT is not yet fully understood, it was reported to be upregulated in MCF10A cells undergoing EMT induced by TGF-β1 stimulation [8]. The roles of the other TFs that our study revealed to undergo early upregulation in EMT, namely *SMARCA1*, *FOXO1*, *ZBTB16*, and *HIPK2*, have yet to be defined. However, unlike the observations related to *ZEB1* and *ZEB2*, which indicate a precise correlation with *VIM* expression, the observation that most other early upregulated TFs exhibited some level of expression, even at the epithelial end (VIM 0–1), does not provide support for the TFs playing a direct causal role in *VIM* upregulation. However, their downregulation upon *ZEB1*-knockdown-mediated EMT reversion substantiates their involvement in acquiring the mesenchymal phenotype throughout the EMT process and suggests a causal role of ZEB1 in their upregulation. The underlying molecular mechanisms of this process warrant further investigation.

In contrast to *ZEB1* and *ZEB2*, *SNAI2* and *TWIST1* exhibited significant upregulation during the mesenchymal end stage of the EMT course. However, the observation of sustained minimal expression of *SNAI2* and consistently high levels of *TWIST1* throughout the early and middle stages of EMT did not provide support for these TFs playing the roles of EMT regulators in this context. In addition to consistent minimal expression of *SNAI1* throughout the EMT process and the absence of enriched expression of those EMT-related TFs in the mesenchymal cluster [4], our findings revealed the context-dependent nature of these EMT-TFs in both EMT-related and non-EMT-related regulation in genetically unperturbed primary breast cancer. Furthermore, our finding indicates that upregulation of core EMT-TFs may not initiate EMT but may be caused by TFs in the EMT cascade under specific conditions. Our observation of *CREB3L1* being enriched in mesenchymal-predominant HM cells through bulk RNA-seq, its significant upregulation at the mesenchymal end of EMT through scRNA-seq, and its lack of EMT reversion upon *CREB3L1* knockdown aligns with this notion. This finding indicates that such TF serves as a marker for the mesenchymal end stage but not as a regulator in the EMT process.

Although we demonstrated the traits and TFs associated with spontaneous EMT, in our culture system, we did not analyze the effect of the tumor microenvironment. However, through in situ RNA-seq, we identified EMT as a prominent gene signature distinguishing Vim^+^ tumor cells from Vim^−^ tumor cells and observed significantly increased expression of *ZEB1*, *ZEB2*, and *CREB3L1* in Vim^+^ tumor cells relative to that in paired Vim^−^ tumor cells within the sampled ROIs in the corresponding primary tumor section. The in vivo data validate our culture system findings, indicating that upregulation of these TFs is intrinsic to carcinoma cells undergoing EMT rather than initiated by the tumor microenvironment [46, 47]. Our discovery, coupled with the finding of a correlation of *VIM* expression with increased NESs of EMT pathways, confirms Vim as a marker for the spontaneous EMT phenotype in breast cancer. This aligns with the poor prognostic effect of Vim expression in breast cancer [31, 48]. In addition, our identification of *HIF1A* upregulation during the EMT process (VIM 4–5) indicates that *HIF1A* upregulation is intrinsic to carcinoma cells undergoing EMT rather than being induced by a hypoxic microenvironment. Although we relied on a single primary carcinoma sample, our finding that gene signatures upregulated in the EMT process in this system correlate with poor patient outcomes validates the biological significance of this model.

In summary, our results confirm the presence of EMP and indicate that genetically unperturbed primary carcinoma undergoing spontaneous EMT may not exhibit a spindle morphology, even in the presence of complete EMT. We illustrated that EMT is a preferred process, whereas MET plays a crucial role in secondary tumor outgrowth. We discovered the TFs that are sequentially and significantly upregulated as carcinoma cells progress through the EMT process and demonstrated that ZEB1 and ZEB2 play pivotal roles in initiating and sustaining the EMT phenotype. Our findings confirm the context-dependent role of core EMT-TFs in EMT. Our results offer insights into the characteristics and TFs of spontaneous EMT in genetically unperturbed primary non-spindled carcinoma.

## Electronic supplementary material

Below is the link to the electronic supplementary material.

Supplementary Material 1: Supplementary Fig. S1 Full uncropped Gels and Blots image of Fig. 1H

Supplementary Material 2: Supplementary Fig. S2 HE and HM lineage establishment and characterization

Supplementary Material 3: Supplementary Fig. S3 Immunofluorescence images of HE-derived single-cell clones

Supplementary Material 4: Supplementary Fig. S4 Phenotypic and functional characterization of HE and HM subclone progenies

Supplementary Material 5: Supplementary Fig. S5 Full uncropped Gels and Blots image of Supplementary Fig. S3

Supplementary Material 6: Supplementary Fig. S6 Full uncropped Gels and Blots image of Fig. 2D

Supplementary Material 7: Supplementary Fig. S7 UMAP plots for TFs reported to be associated with EMT

Supplementary Material 8: Supplementary Fig. S8 UMAP plots of cells stratified by abundance of *VIM* expression into VIM 0–1 (0 < log_2_ expression ≤ 1), VIM 1–2, VIM 2–3, VIM 3–4, VIM 4–5, and VIM 5–6

Supplementary Material 9: Supplementary Fig. S9 Violin plots illustrating expression of mesenchymal markers (*VIM* and *CDH2*), epithelial markers (*CDH1* and *EpCAM*), and *ZEB1* or *ZEB2*, stratified by the expression of *ZEB1* or *ZEB2*

Supplementary Material 10: Supplementary Fig. S10 Violin plots illustrating expression of selected mesenchymal markers (*CDH2*, *FN1*, *VCAN*, *FBN1*, *COL1A2*, *COL6A1*, and *COL6A2*) and epithelial markers (*GRHL2*, *CDH1*, *EpCAM*, *TJP2*, *CLDN7*, *KRT8*, *KRT18*, *CDH3*, and *JUP*) stratified by *VIM* expression

Supplementary Material 11: Supplementary Fig. S11 UMAP of differentially upregulated TFs (*p* < 0.05, log_2_FC ≥ 1) between each VIM subgroup and VIM 0–1 subgroup

Supplementary Material 12: Supplementary Fig. S12 Violin plot illustrating expression of *ZEB1*, *ZEB2*, *CREB3L1*, *SNAI1*, *SNAI2*, and *TWIST1* stratified by *VIM* expression

Supplementary Material 13: Supplementary Fig. S13 Violin plot illustrating expression of selected mesenchymal markers *VIM*, *CDH2* and *FN1* and epithelial markers *CDH1* and *EpCAM* stratified by the expression of core EMT-regulators *ZEB1*, *ZEB2*, *SNAI1*, *SNAI2*, and *TWIST1*

Supplementary Material 14: Supplementary Fig. S14 Correlation of ZEB1, ZEB2, and Slug with Vim expression in the MpBC cohort. (A) Bar plots illustrating the positivity ratio of Vim and ZEB1, ZEB2, and Slug staining in the conventional ductal carcinoma (NST) components and spindle carcinomatous (SPS) components in the MpBC cases with paired NST and SPS components. (B) IHC staining for Vim, ZEB1, ZEB2, and Slug in paired NST and SPS components in three representative cases of MpBC. (Magnification, 200×)

Supplementary Material 15: Supplementary Fig. S15 Differences in expression of *SNAI1*, *SNAI2*, and *TWIST1* between paired Vim^+^ and Vim^−^ tumor cells from the nine ROIs are plotted, with the paired *t* test used for testing these differences

Supplementary Material 16: Supplementary Table S1 Reagents and antibodies used in this study

Supplementary Material 17: Supplementary Table S2 Primer sequences used in qRT-PCR

Supplementary Material 18: Supplementary Table S3 RNA-seq results (TPM) for top enriched TFs in the M (HM, M-sc1, and M-sc5) and E (HE, E-sc60, and E-sc66) groups

Supplementary Material 19: Supplementary Table S4 Rank correlation between abundance of vimentin expression and 50 Hallmark signaling pathways

Supplementary Material 20: Supplementary Table S5 Differentially significantly upregulated TFs (*p* < 0.05, log2FC ≥ 1) between each VIM subgroup versus VIM 0–1 subgroup

## Data Availability

Sequence data that support the findings of this study have been deposited in theGene Expression Omnibus (GEO; www.ncbi.nlm.nih.gov/geo/; number: GSE261037; secure token: kzarwssmftghbgt; GSE183905; secure token: abkbimucpdojtqz).

## Abbreviations

AOI: Area of illumination
bHLH: Basic helix-loop-helix
CDH1: Cadherin 1
CDH2: Cadherin 2
CITED2: Cbp/P300 Interacting Transactivator With Glu/Asp Rich Carboxy-Terminal Domain 2
CK: Cytokeratin
CREB3: CAMP Responsive Element Binding Protein 3
CREB3L1: CAMP Responsive Element Binding Protein 3 Like 1
DAB: 3,3’-Diaminobenzidine
DAPI: 4’, 6-diamidino-2-phenylindole
DEG: Differentially expressed gene
DFI: Disease-free interval
DSP: Digital spatial profiler
E-cad: E-cadherin
EMP: Epithelial-mesenchymal plasticity
EMT: Epithelial-mesenchymal transition
EpCAM: Epithelial cell adhesion molecule
FFPE: Formalin-fixed paraffin-embedded
FOXO1: Forkhead box protein O1
GAPDH: Glyceraldehyde 3-phosphate dehydrogenase
GDC: Genomic data commons
GSEA: Gene set enrichment analysis
GSVA: Gene set variation analysis
HIPK2: Homeodomain Interacting Protein Kinase 2
HLX: H2.0-like homeobox protein
ID1: Inhibitor of DNA binding 1
ID3: Inhibitor of DNA binding 3
IHC: Immunohistochemistry
MAD: Median absolute deviation
MET: Mesenchymal-epithelial transition
MMSCC: Monophasic metaplastic squamous cell carcinoma
MpBC: Metaplastic breast carcinoma
MSigDB: Molecular signatures database
MXD4: MAX Dimerization Protein 4
NES: Normalized enrichment scores
NFKB1A: Nuclear Factor Kappa B Subunit 1
NST: No special type
OS: Overall survival
PanCK: Pan-cytokeratin
PCA: Principal component analysis
PCR: Polymerase chain reaction
PD-L1: Programmed cell death 1 ligand 1
qRT-PCR: Quantitative reverse transcription polymerase chain reaction
RNA-seq: RNA sequencing
ROI: Region of interest
scRNA-seq: Single cell RNA sequencing
shRNA: Short hairpin RNA
SMARCA1: SWI/SNF related, matrix associated, actin dependent regulator of chromatin, subfamily a, member 1
SNAI1: Snail family transcriptional repressor 1
SNAI2: Snail family transcriptional repressor 2
SPS: Spindle carcinomatous
TF: Transcription factor
TGF-β1: Transforming growth factor-β1
TNBC: Triple-negative breast cancer
TRRUST: Transcriptional regulatory relationships unravelled by sentence-based text-mining
TWIST1: Twist family bHLH transcription factor 1
UMAP: Uniform manifold approximation and projection
VIM: Vimentin
ZBTB16: Zinc Finger And BTB Domain Containing 16
ZEB1: Zinc finger E-box binding homeobox 1
ZEB2: Zinc finger E-box binding homeobox 2
ZNF300: Zinc finger protein 300
