# Supplementary figures and images for: Characteristics and transcriptional regulators of spontaneous epithelial–mesenchymal transition in genetically unperturbed patient-derived non-spindled breast carcinoma
Source: Breast Cancer Res. 2024 Sep 10;26:130. doi: 10.1186/s13058-024-01888-5 (PMC11385830; doi:10.1186/s13058-024-01888-5)

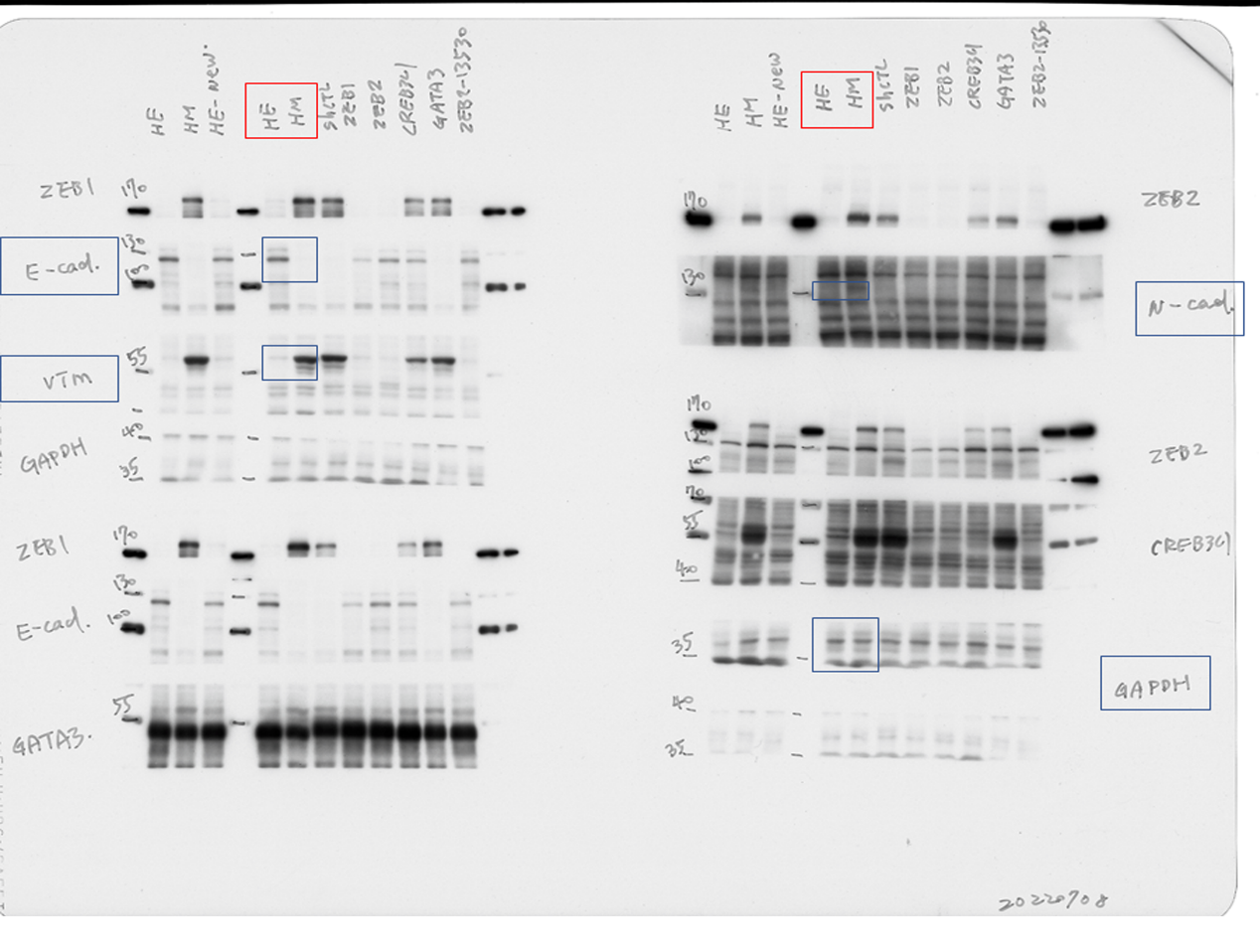


**Supplementary Fig. S1** Full uncropped Gels and Blots image of Fig. 1H

Supplement: Supplementary file 1 — Supplementary Material 1: Supplementary Fig. S1 Full uncropped Gels and Blots image of Fig. 1H [file 13058_2024_1888_MOESM1_ESM.docx]

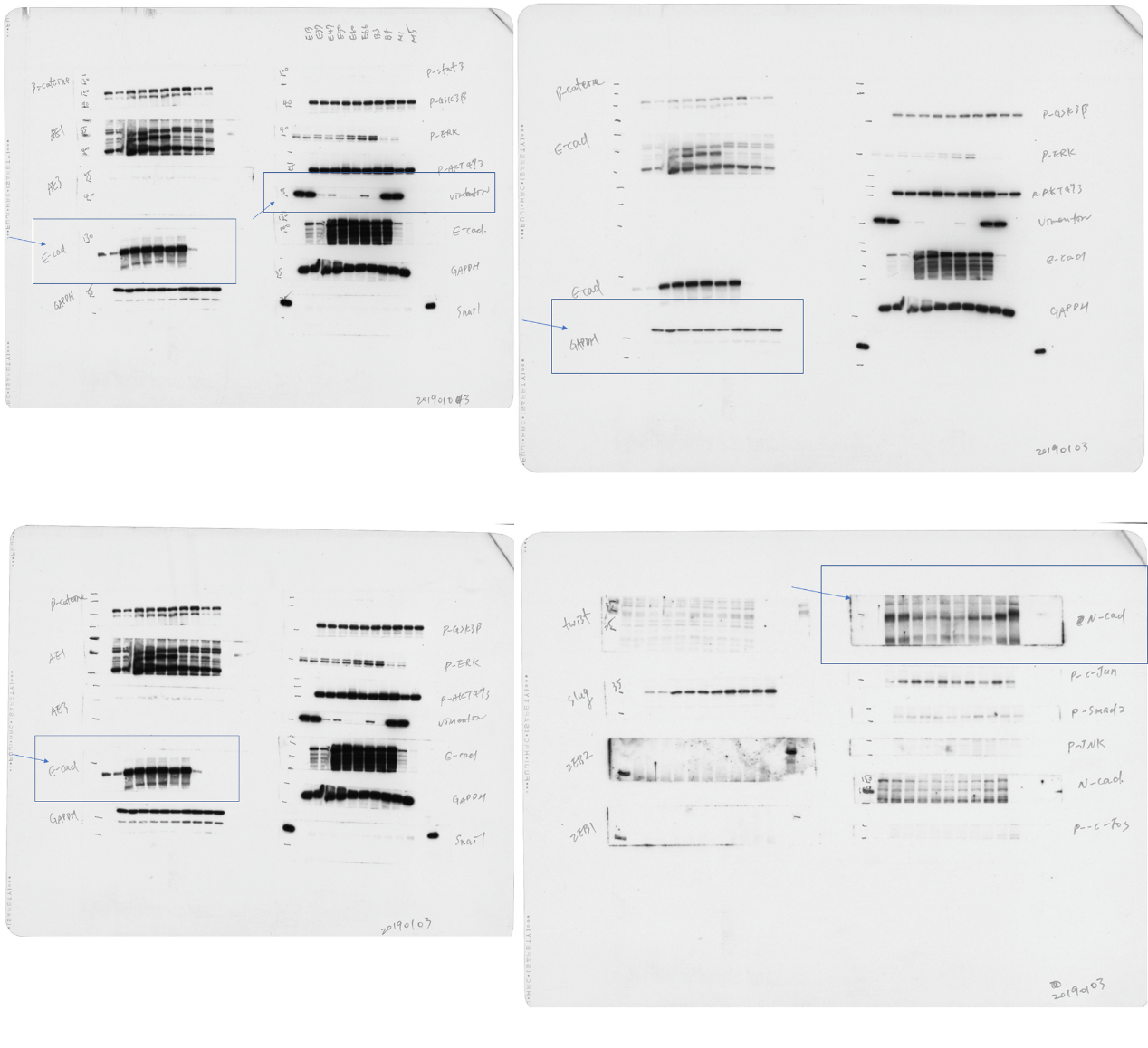


**Supplementary Fig. S5** Full uncropped Gels and Blots image of Supplementary Fig. S3

Supplement: Supplementary file 5 — Supplementary Material 5: Supplementary Fig. S5 Full uncropped Gels and Blots image of Supplementary Fig. S3 [file 13058_2024_1888_MOESM5_ESM.docx]

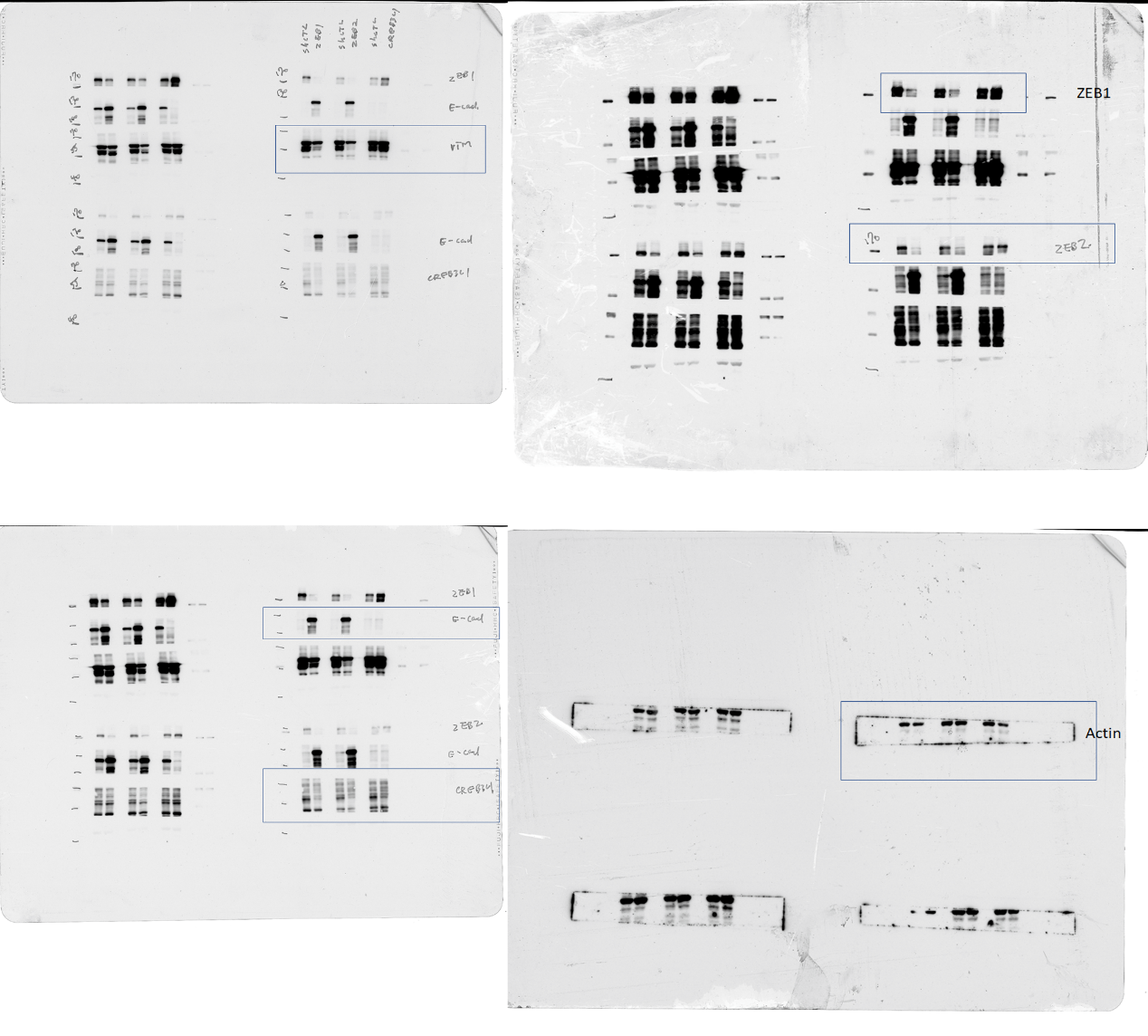


**Supplementary Fig. S6** Full uncropped Gels and Blots image of Fig. 2D

Supplement: Supplementary file 6 — Supplementary Material 6: Supplementary Fig. S6 Full uncropped Gels and Blots image of Fig. 2D [file 13058_2024_1888_MOESM6_ESM.docx]

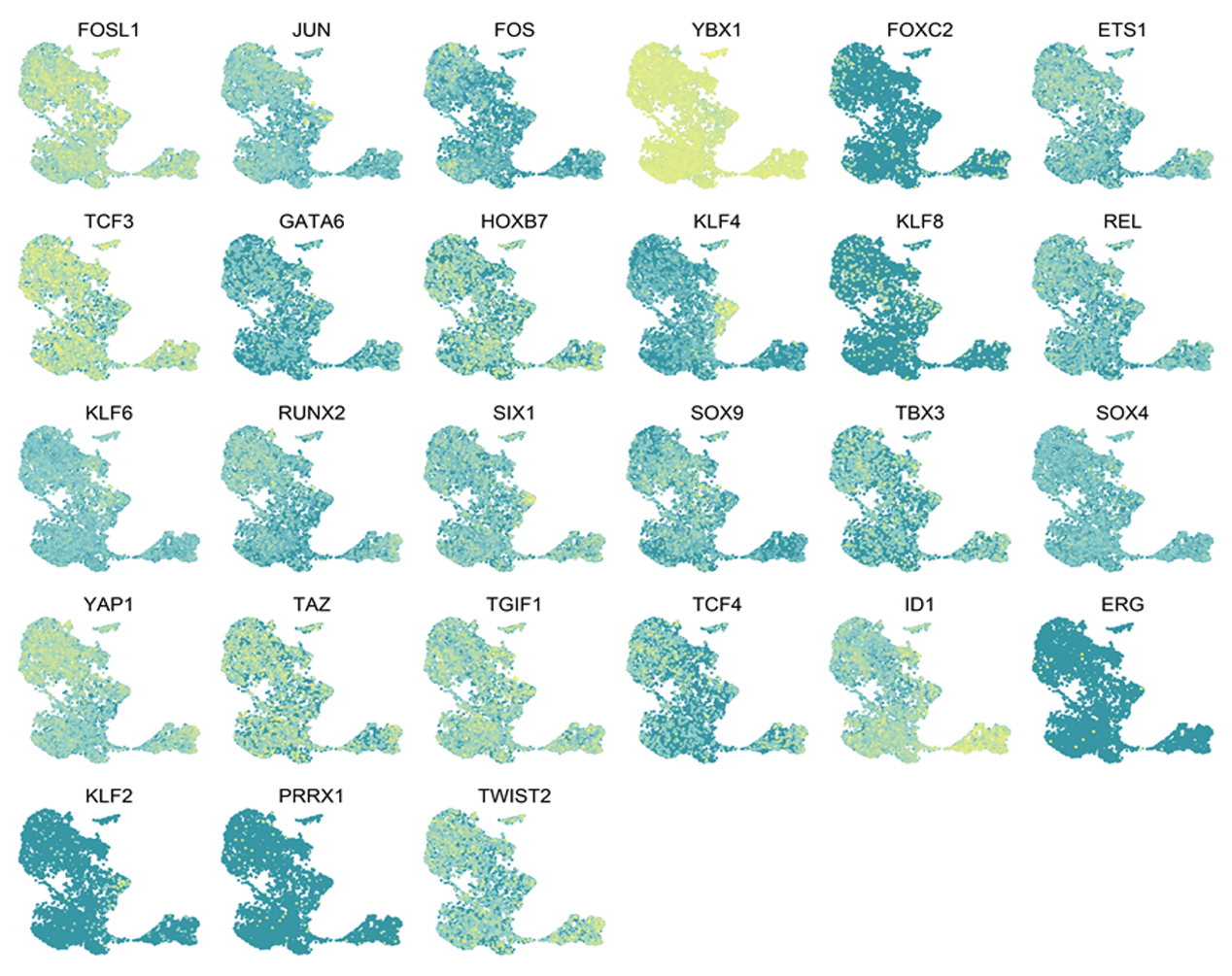


**Supplementary Fig. S7** UMAP plots for TFs reported to associate with EMT.

Supplement: Supplementary file 7 — Supplementary Material 7: Supplementary Fig. S7 UMAP plots for TFs reported to be associated with EMT [file 13058_2024_1888_MOESM7_ESM.docx]
