# Supplementary material for: Characteristics and transcriptional regulators of spontaneous epithelial–mesenchymal transition in genetically unperturbed patient-derived non-spindled breast carcinoma
Source: Breast Cancer Res. 2024 Sep 10;26:130. doi: 10.1186/s13058-024-01888-5 (PMC11385830; doi:10.1186/s13058-024-01888-5)
Supplement: Supplementary file 2 — Supplementary Material 2: Supplementary Fig. S2 HE and HM lineage establishment and characterization [file 13058_2024_1888_MOESM2_ESM.docx]

**
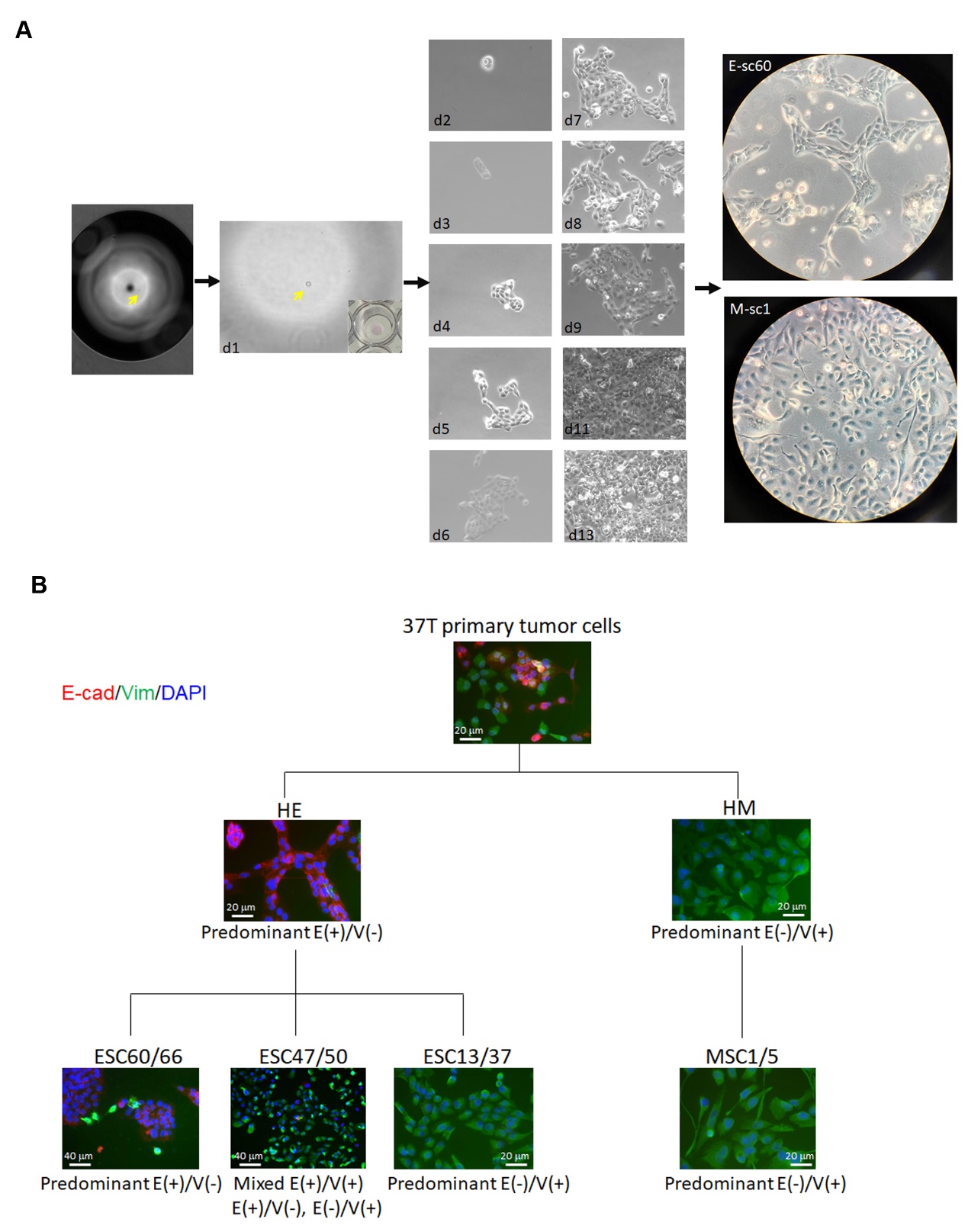
**

**Supplementary Fig. S2** HE and HM subclone progenies establishment and characterization. **A** Illustration of a single-cell cloning process. The cells were diluted to 2000 cells per 10-cm dish and then allowed to sediment for 10 min; single cells were then picked up one by one with a 10-μL pipette tip under a light microscope (arrow; first left panel). These cells were then transferred to 96-well plates. We then observed them under light microscopy, and wells with only one cell (arrow, second left panel) were filled with medium and incubated in a CO_2_ atmosphere. The cultivation process was monitored daily and representative phase-contrast images taken from indicated days of culture are shown in the middle panel. Representative established HE subclone E-sc60 and HM subclone M-sc1 are shown in the right panel. **B** Pedigree of representative HE and HM subclones classified on the basis of immunofluorescence staining for E-cad (E) and Vim (V). HE subclones included epithelial-predominant subclones (e.g. E-sc60 and E-sc66), mixed epithelial and mesenchymal subclones (e.g. E-sc47 and E-sc50), and mesenchymal-predominant subclones (e.g. E-sc13 and E-sc37), whereas HM subclones were solely composed of mesenchymal-predominant subclones (e.g. M-sc1 and M-sc5).
