# Supplementary material for: Characteristics and transcriptional regulators of spontaneous epithelial–mesenchymal transition in genetically unperturbed patient-derived non-spindled breast carcinoma
Source: Breast Cancer Res. 2024 Sep 10;26:130. doi: 10.1186/s13058-024-01888-5 (PMC11385830; doi:10.1186/s13058-024-01888-5)
Supplement: Supplementary file 3 — Supplementary Material 3: Supplementary Fig. S3 Immunofluorescence images of HE-derived single-cell clones [file 13058_2024_1888_MOESM3_ESM.docx]

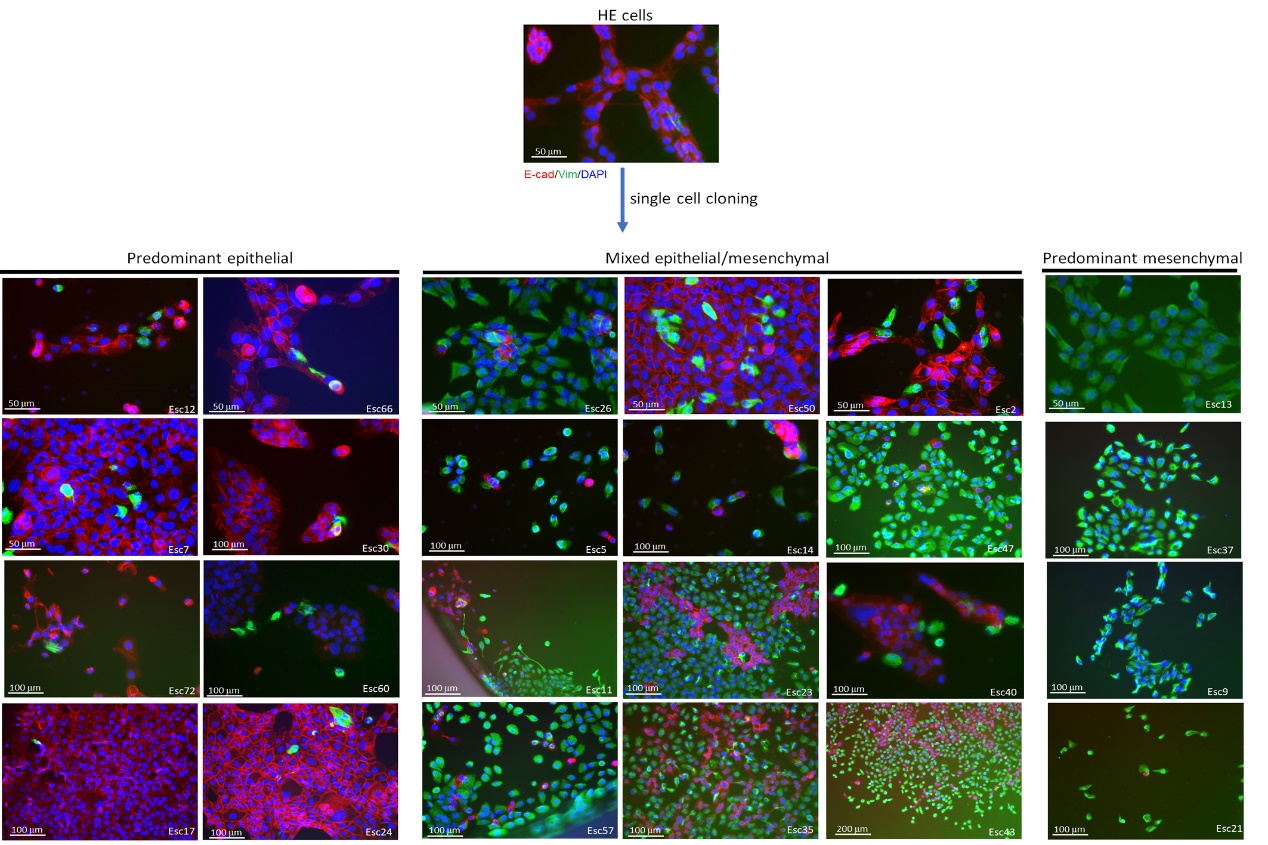


**Supplementary Fig. S3** Immunofluorescence images of HE-derived single-cell clones. The immunofluorescence images of Esc11 and Esc57 were taken directly from the 12-well dish during analysis.
