# Supplementary material for: Characteristics and transcriptional regulators of spontaneous epithelial–mesenchymal transition in genetically unperturbed patient-derived non-spindled breast carcinoma
Source: Breast Cancer Res. 2024 Sep 10;26:130. doi: 10.1186/s13058-024-01888-5 (PMC11385830; doi:10.1186/s13058-024-01888-5)
Supplement: Supplementary file 4 — Supplementary Material 4: Supplementary Fig. S4 Phenotypic and functional characterization of HE and HM subclone progenies [file 13058_2024_1888_MOESM4_ESM.docx]

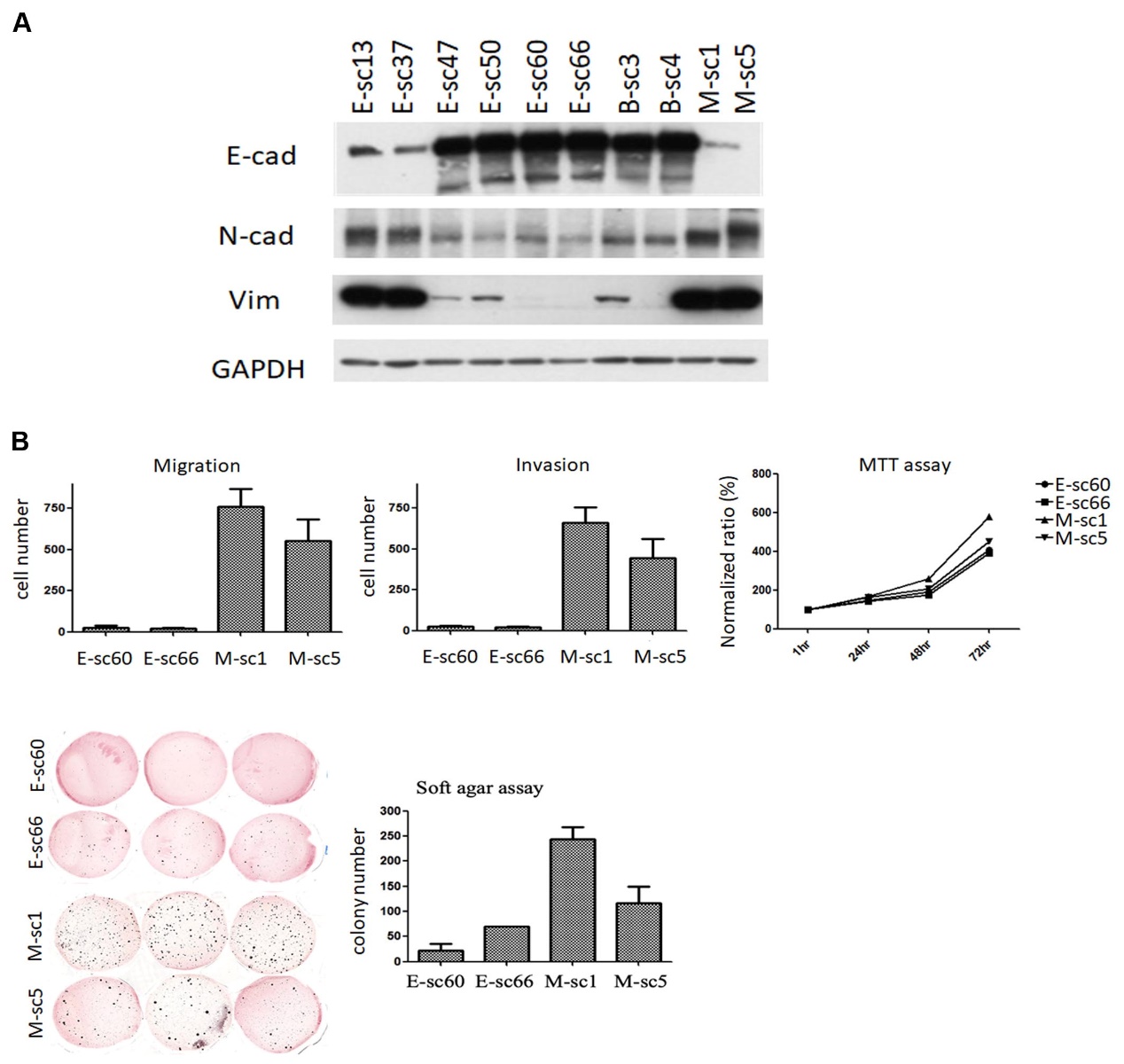


**Supplementary Fig. S4** Phenotypic and functional characterization of HE and HM subclone progenies. **A** Western blotting for E-cad and Vim expression in HE and HM subclones, including epithelial-predominant subclones (E-sc60 and E-sc66), mixed epithelial and mesenchymal subclones (E-sc47 and E-sc50), mesenchymal-predominant subclones (E-sc13 and E-sc37), and mesenchymal-predominant HM subclones (M-sc1 and M-sc5). Two additional clones, B-sc3 and B-sc4, which showed variable E-cad and Vim expression, were not included in the subsequent analyses. **B** Transwell and soft agar assays. The migration and invasion ability were examined using transwells without and with Matrigel coating, respectively, and anchorage-independent growth ability was examined using soft agar assay. The MTT assay was used to measure cell proliferation. The OD value of each cell was normalised by the corresponding OD value at 1 h.
