# Supplementary material for: Characteristics and transcriptional regulators of spontaneous epithelial–mesenchymal transition in genetically unperturbed patient-derived non-spindled breast carcinoma
Source: Breast Cancer Res. 2024 Sep 10;26:130. doi: 10.1186/s13058-024-01888-5 (PMC11385830; doi:10.1186/s13058-024-01888-5)
Supplement: Supplementary file 8 — Supplementary Material 8: Supplementary Fig. S8 UMAP plots of cells stratified by abundance of VIM expression into VIM 0–1 (0 < log2 expression ≤ 1), VIM 1–2, VIM 2–3, VIM 3–4, VIM 4–5, and VIM 5–6 [file 13058_2024_1888_MOESM8_ESM.docx]

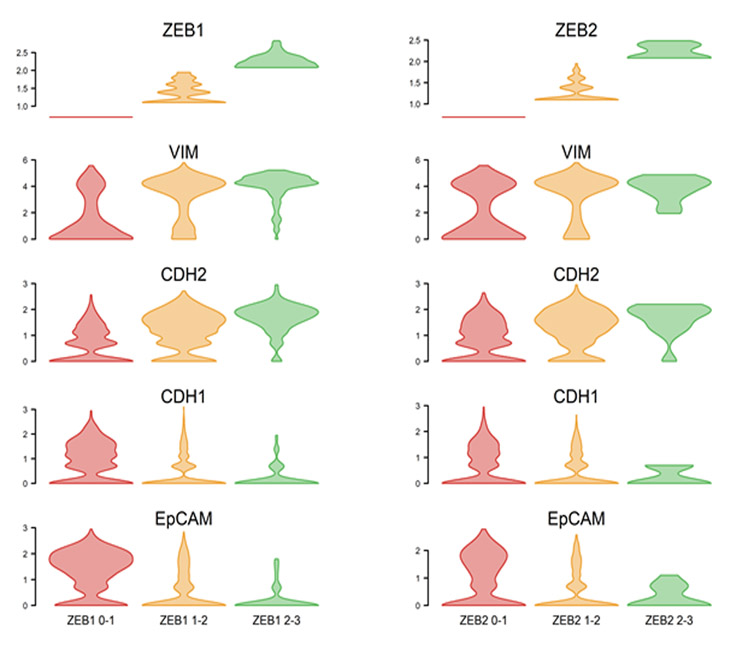


**Supplementary Fig. S8** Violin plots illustrating expression of mesenchymal markers (*VIM* and *CDH2*), epithelial markers (*CDH1* and *EpCAM*), and *ZEB1* or *ZEB2,* stratified by the expression of *ZEB1* (left) or *ZEB2* (right).
