# Supplementary material for: Characteristics and transcriptional regulators of spontaneous epithelial–mesenchymal transition in genetically unperturbed patient-derived non-spindled breast carcinoma
Source: Breast Cancer Res. 2024 Sep 10;26:130. doi: 10.1186/s13058-024-01888-5 (PMC11385830; doi:10.1186/s13058-024-01888-5)
Supplement: Supplementary file 9 — Supplementary Material 9: Supplementary Fig. S9 Violin plots illustrating expression of mesenchymal markers (VIM and CDH2), epithelial markers (CDH1 and EpCAM), and ZEB1 or ZEB2, stratified by the expression of ZEB1 or ZEB2 [file 13058_2024_1888_MOESM9_ESM.docx]

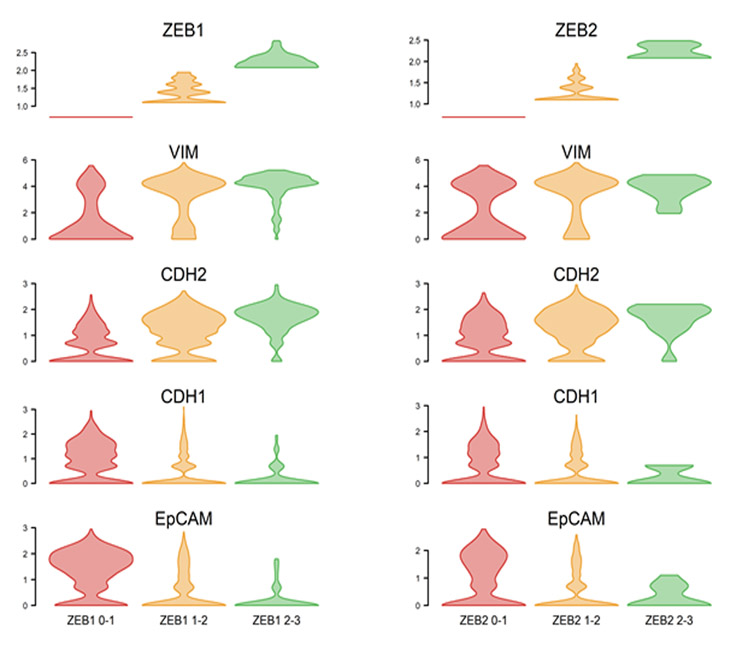


**Supplementary Fig. S9** Violin plots illustrating expression of mesenchymal markers (*VIM* and *CDH2*), epithelial markers (*CDH1* and *EpCAM*), and *ZEB1* or *ZEB2,* stratified by the expression of *ZEB1* (left) or *ZEB2* (right).
