# Supplementary material for: Characteristics and transcriptional regulators of spontaneous epithelial–mesenchymal transition in genetically unperturbed patient-derived non-spindled breast carcinoma
Source: Breast Cancer Res. 2024 Sep 10;26:130. doi: 10.1186/s13058-024-01888-5 (PMC11385830; doi:10.1186/s13058-024-01888-5)
Supplement: Supplementary file 10 — Supplementary Material 10: Supplementary Fig. S10 Violin plots illustrating expression of selected mesenchymal markers (CDH2, FN1, VCAN, FBN1, COL1A2, COL6A1, and COL6A2) and epithelial markers (GRHL2, CDH1, EpCAM, TJP2, CLDN7, KRT8, KRT18, CDH3, and JUP) stratified by VIM expression [file 13058_2024_1888_MOESM10_ESM.docx]

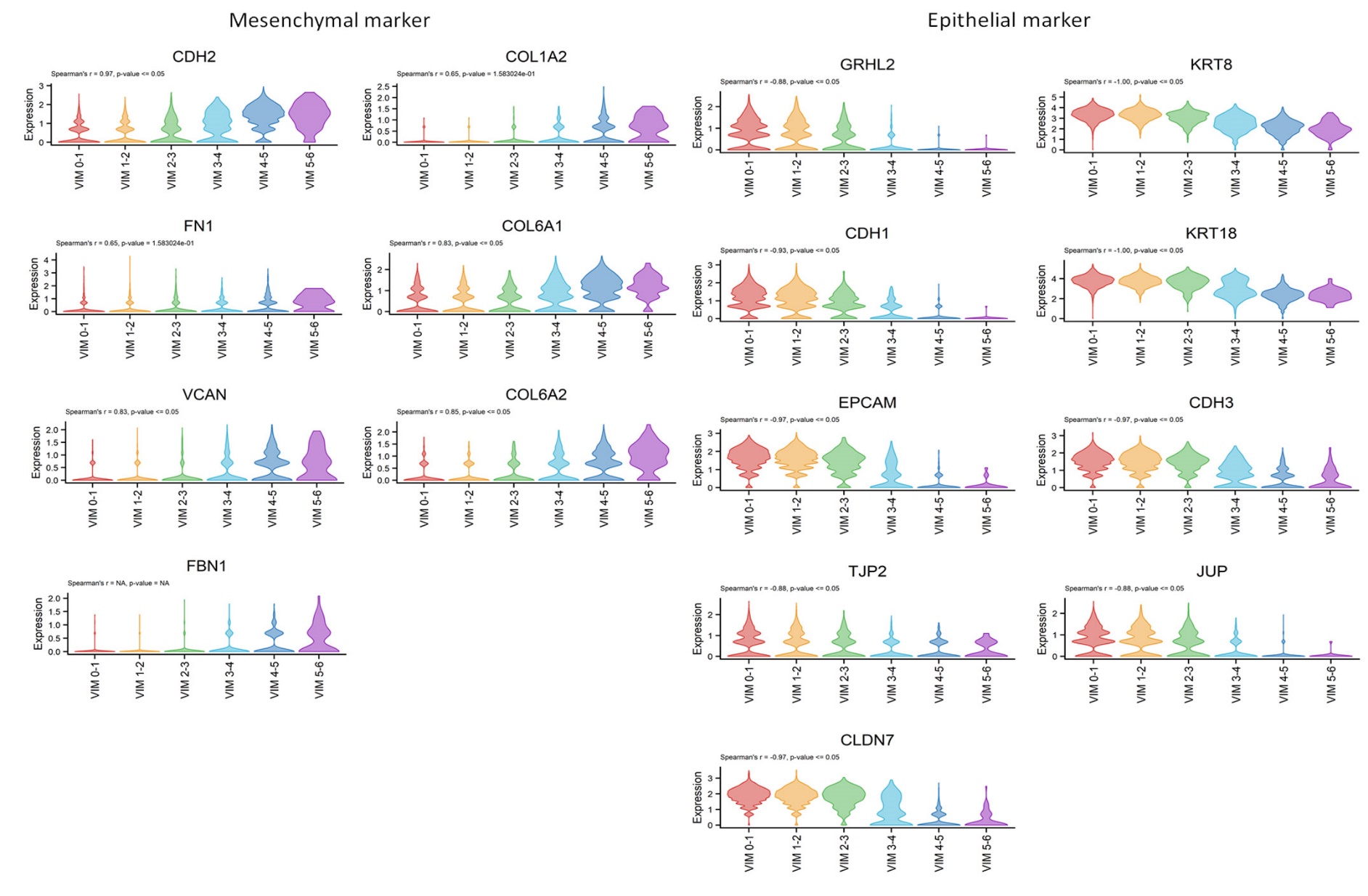


**Supplementary Fig. S10** Violin plots illustrating expression of selected mesenchymal markers (*CDH2*, *FN1*, *VCAN*, *FBN1*, *COL1A2*, *COL6A1*, and *COL6A2*) and epithelial markers (*GRHL2*, *CDH1*, *EpCAM*, *TJP2*, *CLDN7*, *KRT8*, *KRT18*, *CDH3*, and *JUP*) stratified by *VIM* expression.
