# Supplementary material for: Characteristics and transcriptional regulators of spontaneous epithelial–mesenchymal transition in genetically unperturbed patient-derived non-spindled breast carcinoma
Source: Breast Cancer Res. 2024 Sep 10;26:130. doi: 10.1186/s13058-024-01888-5 (PMC11385830; doi:10.1186/s13058-024-01888-5)
Supplement: Supplementary file 11 — Supplementary Material 11: Supplementary Fig. S11 UMAP of differentially upregulated TFs (p < 0.05, log2FC ≥ 1) between each VIM subgroup and VIM 0–1 subgroup [file 13058_2024_1888_MOESM11_ESM.docx]

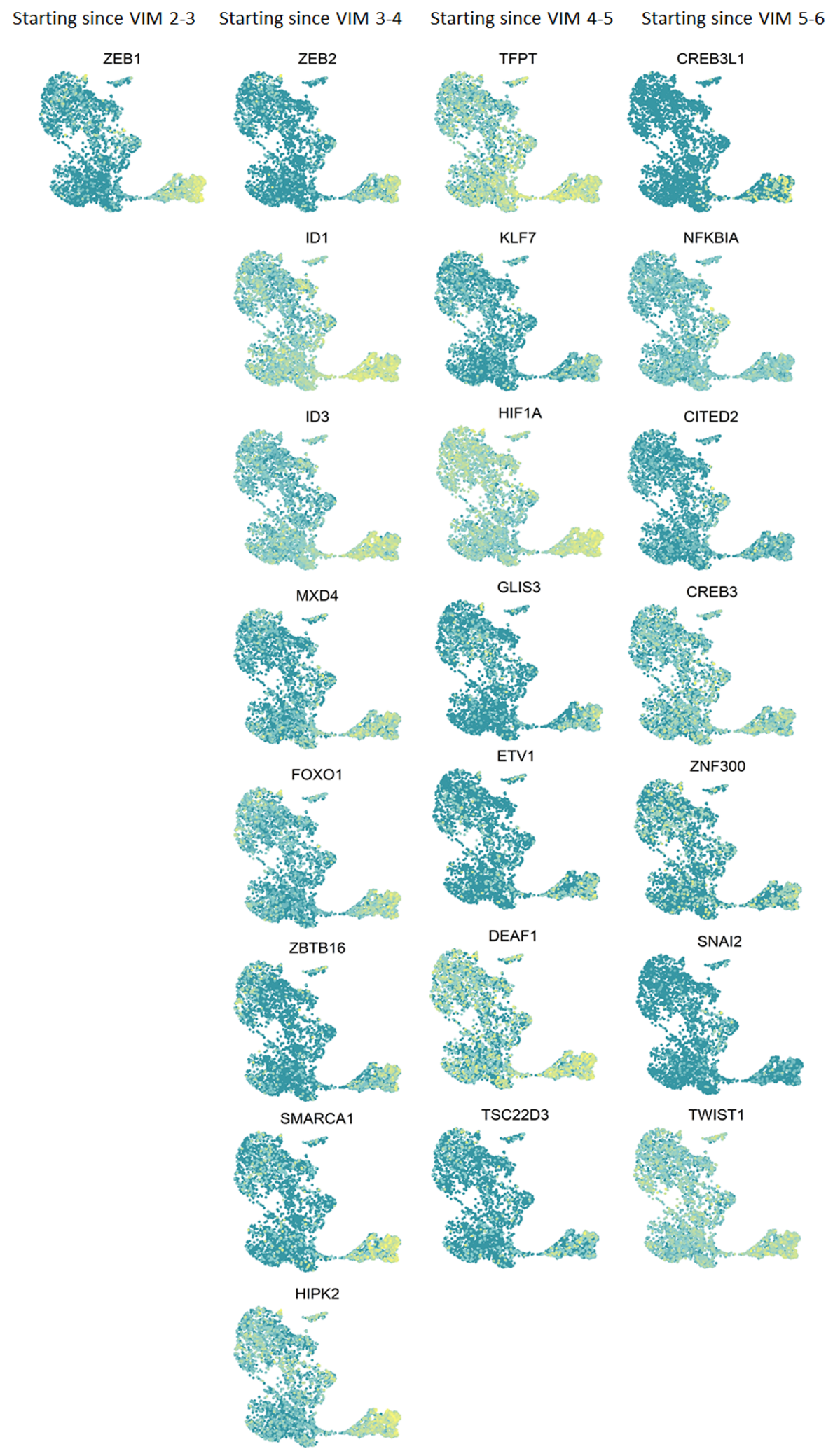


**Supplementary Fig. S11** UMAP plots illustrating differentially significantly upregulated TFs (*p* < 0.05, log2FC ≧1) between each VIM subgroup and the VIM 0-1 subgroup
