# Supplementary material for: Characteristics and transcriptional regulators of spontaneous epithelial–mesenchymal transition in genetically unperturbed patient-derived non-spindled breast carcinoma
Source: Breast Cancer Res. 2024 Sep 10;26:130. doi: 10.1186/s13058-024-01888-5 (PMC11385830; doi:10.1186/s13058-024-01888-5)
Supplement: Supplementary file 12 — Supplementary Material 12: Supplementary Fig. S12 Violin plot illustrating expression of ZEB1, ZEB2, CREB3L1, SNAI1, SNAI2, and TWIST1 stratified by VIM expression [file 13058_2024_1888_MOESM12_ESM.docx]

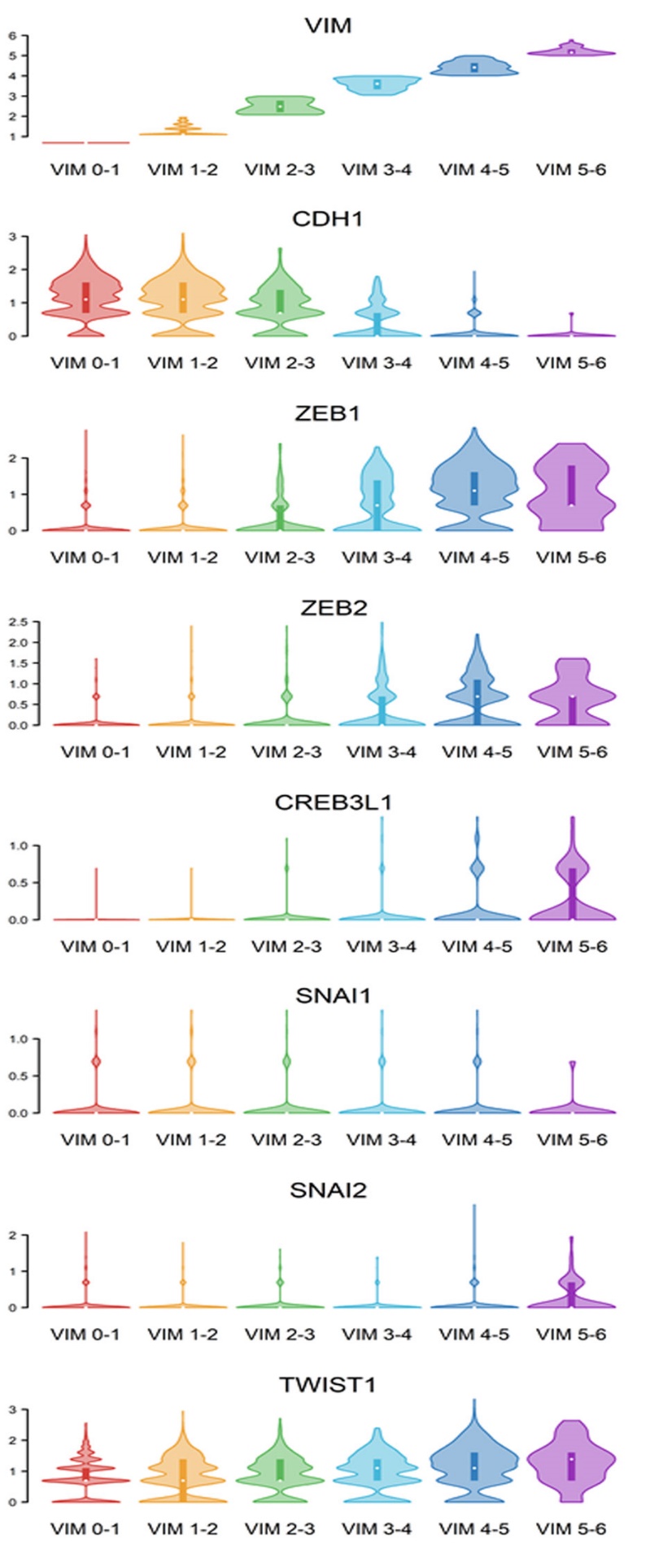


**Supplementary Fig. S12** Violin plot illustrating expression of *ZEB1*, *ZEB2*, *CREB3L1*, *SNAI1*, *SNAI2*, and *TWIST1* stratified by *VIM* expression.
