# Supplementary material for: Characteristics and transcriptional regulators of spontaneous epithelial–mesenchymal transition in genetically unperturbed patient-derived non-spindled breast carcinoma
Source: Breast Cancer Res. 2024 Sep 10;26:130. doi: 10.1186/s13058-024-01888-5 (PMC11385830; doi:10.1186/s13058-024-01888-5)
Supplement: Supplementary file 13 — Supplementary Material 13: Supplementary Fig. S13 Violin plot illustrating expression of selected mesenchymal markers VIM, CDH2 and FN1 and epithelial markers CDH1 and EpCAM stratified by the expression of core EMT-regulators ZEB1, ZEB2, SNAI1, SNAI2, and TWIST1 [file 13058_2024_1888_MOESM13_ESM.docx]

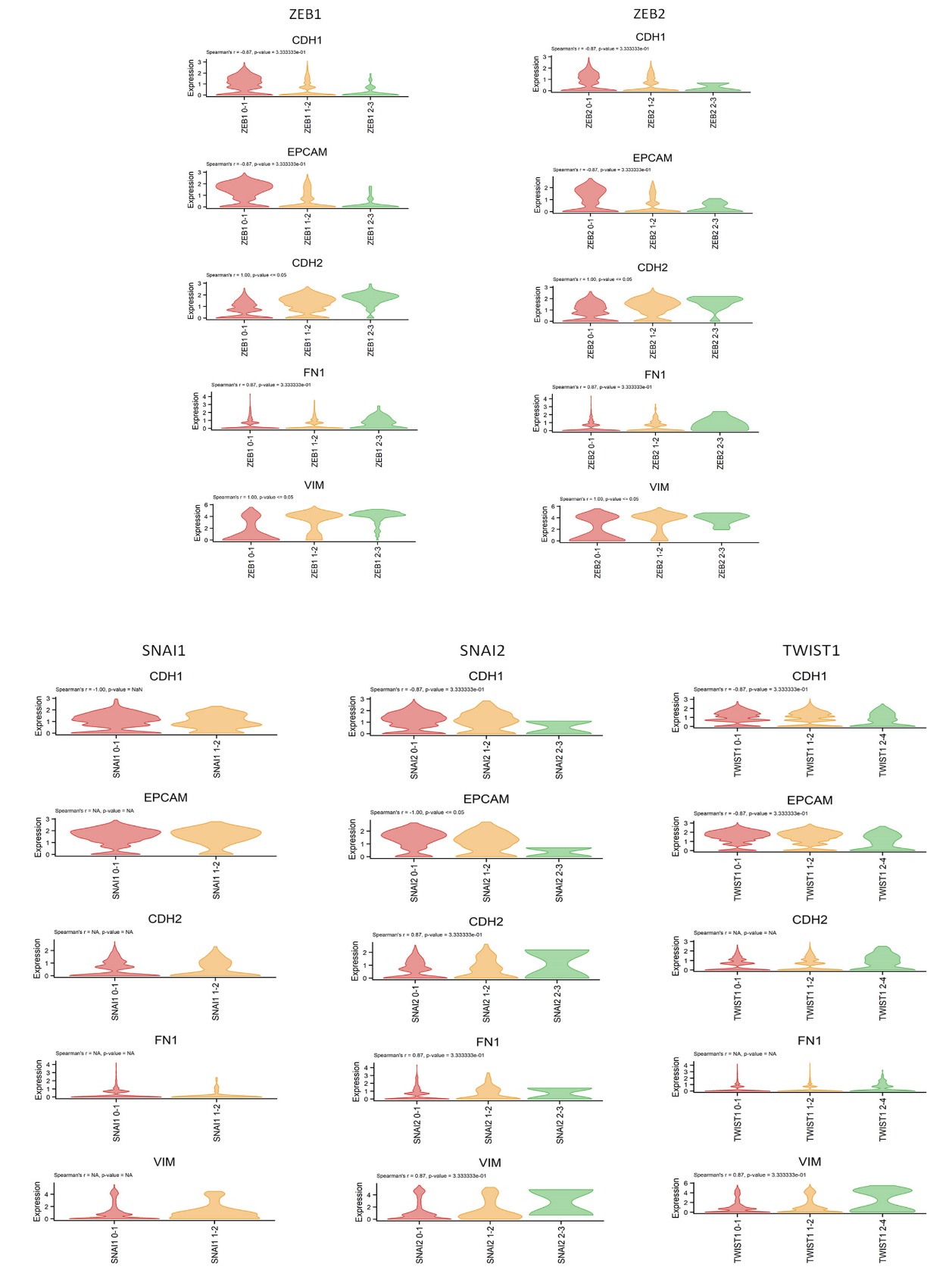


**Supplementary Fig. S13** Violin plot illustrating expression of selected mesenchymal markers *VIM*, *CDH2* and *FN1* and epithelial markers *CDH1* and *EpCAM* stratified by the expression of core EMT-regulators *ZEB1*, *ZEB2*, *SNAI1*, *SNAI2*, and *TWIST1*.
