# Supplementary material for: Characteristics and transcriptional regulators of spontaneous epithelial–mesenchymal transition in genetically unperturbed patient-derived non-spindled breast carcinoma
Source: Breast Cancer Res. 2024 Sep 10;26:130. doi: 10.1186/s13058-024-01888-5 (PMC11385830; doi:10.1186/s13058-024-01888-5)
Supplement: Supplementary file 14 — Supplementary Material 14: Supplementary Fig. S14 Correlation of ZEB1, ZEB2, and Slug with Vim expression in the MpBC cohort. (A) Bar plots illustrating the positivity ratio of Vim and ZEB1, ZEB2, and Slug staining in the conventional ductal carcinoma (NST) components and spindle carcinomatous (SPS) components in the MpBC cases with paired NST and SPS components. (B) IHC staining for Vim, ZEB1, ZEB2, and Slug in paired NST and SPS components in three representative cases of MpBC. (Magnification, 200×) [file 13058_2024_1888_MOESM14_ESM.docx]

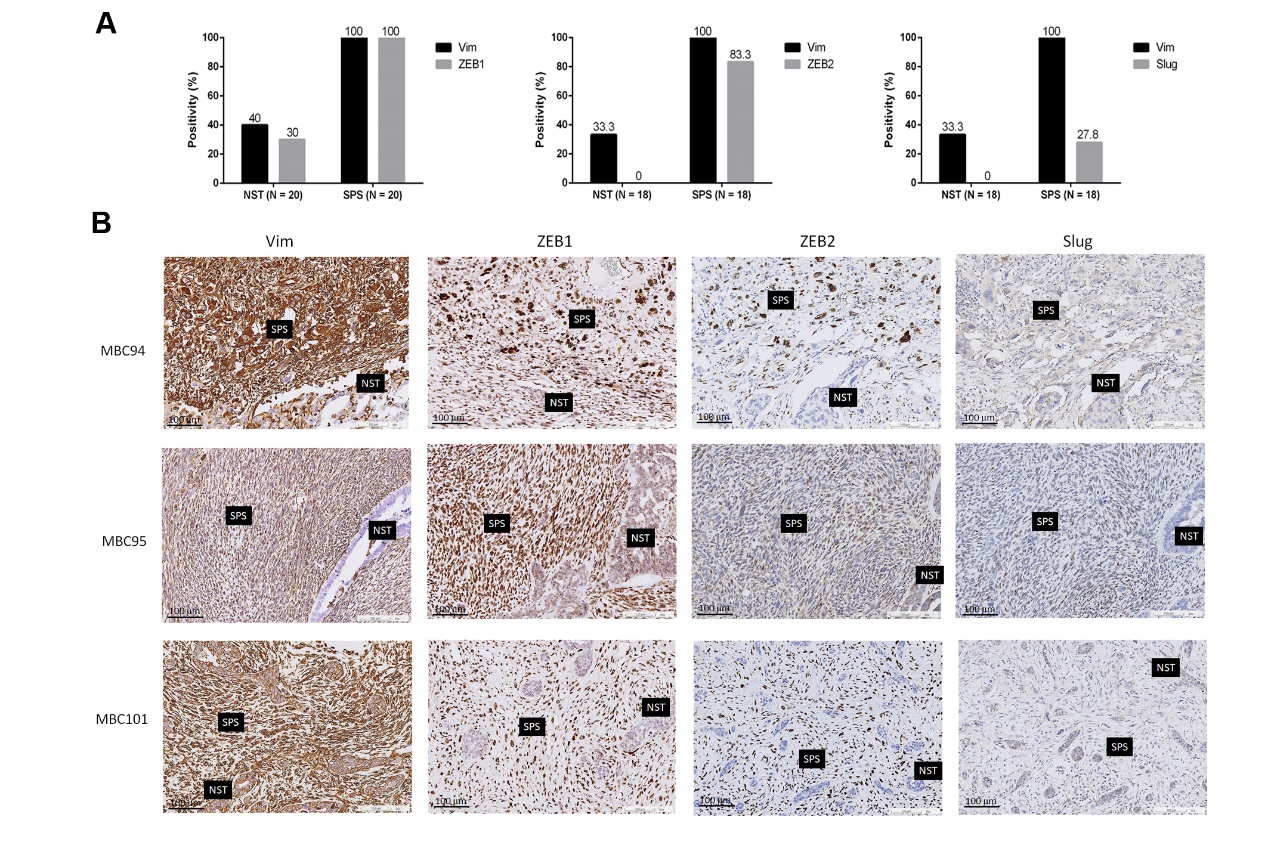


**Supplementary Fig. S14** Correlation of ZEB1, ZEB2, and Slug with Vim expression in the MpBC cohort. **A.** Bar plots illustrating the positivity ratio of Vim and ZEB1, ZEB2, and Slug staining in the conventional ductal carcinoma (NST) components and spindle carcinomatous (SPS) components in the MpBC cases with paired NST and SPS components. **B.** IHC staining for Vim, ZEB1, ZEB2, and Slug was shown in three representative MpBC cases (MBC94, MBC95, and MBC101). Staining for Vim, ZEB1, and ZEB2 was observed in the SPS components of all three cases. Slug staining was present in the SPS component of MBC95 but was absent in MBC94 and MBC101.
