# Supplementary material for: Characteristics and transcriptional regulators of spontaneous epithelial–mesenchymal transition in genetically unperturbed patient-derived non-spindled breast carcinoma
Source: Breast Cancer Res. 2024 Sep 10;26:130. doi: 10.1186/s13058-024-01888-5 (PMC11385830; doi:10.1186/s13058-024-01888-5)
Supplement: Supplementary file 15 — Supplementary Material 15: Supplementary Fig. S15 Differences in expression of SNAI1, SNAI2, and TWIST1 between paired Vim+ and Vim− tumor cells from the nine ROIs are plotted, with the paired t test used for testing these differences [file 13058_2024_1888_MOESM15_ESM.docx]

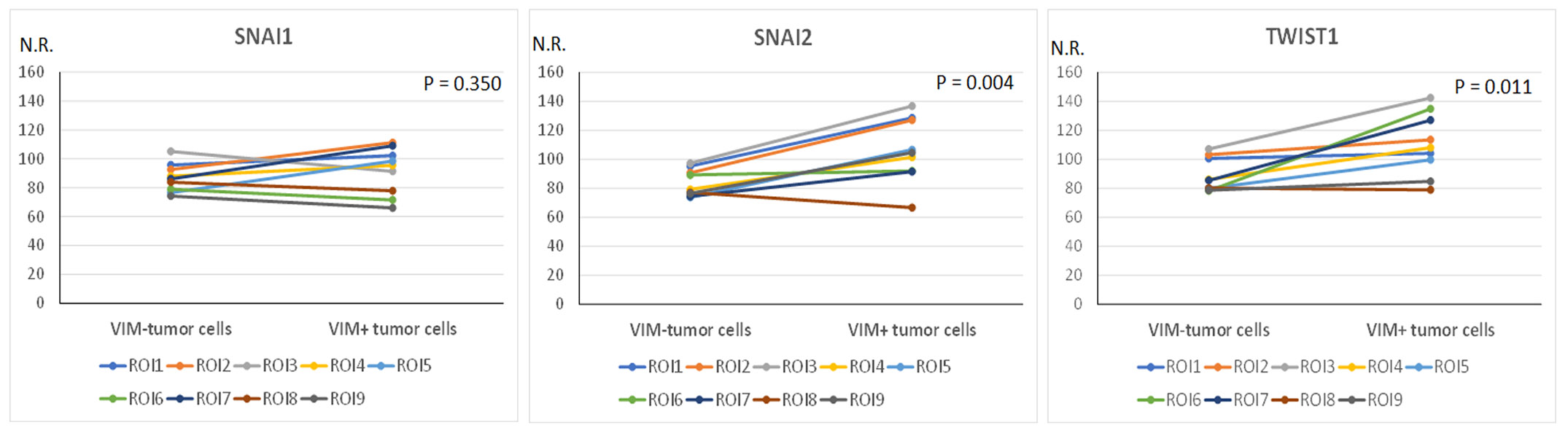


**Supplementary Fig. S15** Differences in expression of *SNAI1*, *SNAI2*, and *TWIST1* between paired Vim^+^ and Vim^−^ tumor cells from the nine ROIs are plotted, with the paired *t* test used for testing these differences.
