# Supplementary material for: Characteristics and transcriptional regulators of spontaneous epithelial–mesenchymal transition in genetically unperturbed patient-derived non-spindled breast carcinoma
Source: Breast Cancer Res. 2024 Sep 10;26:130. doi: 10.1186/s13058-024-01888-5 (PMC11385830; doi:10.1186/s13058-024-01888-5)
Supplement: Supplementary file 16 — Supplementary Material 16: Supplementary Table S1 Reagents and antibodies used in this study [file 13058_2024_1888_MOESM16_ESM.docx]

**Supplementary Table S1** Reagents and antibodies used in this study

| Reagents |  |
| --- | --- |
| Storing reagent |  |
|  | cellbanker 1 reagent (ZENOGEN PHARMA, Fukushima, Jpn), |
| Digestion medium |  |
|  | collagenase type III (0.1%) (WT-LS004182, Worthington Biochemical Corp., Lakewood, NJ, USA) |
|  | Dispase (40ul/ml) (17105-041, Gibco, Waltham, MA, USA) |
| Culture medium |  |
|  | DMEM/F12 (10565-018, Gibco) |
|  | FBS (2%) (2198255RP, Gibco) |
|  | NEAA (1%) (11140035, Gibco) |
|  | sodium pyruvate (1mM) (11360070, Gibco) |
|  | Nicotinamide (1mM) (N0636, Sigma-Aldrich, Inc., St. Louis, MO, USA) |
|  | insulin (20 ug/ml) (51500-056, Gibco) |
|  | hydrocortisone (0.5 ug/ml) (H0888, Sigma-Aldrich, Inc.) |
|  | EGF (10 ng/ml) (585506, BioLegend, San Diego, CA, USA) |
|  | ascorbic acid (200 uM) (A8960, Sigma-Aldrich, Inc.) |
|  | adenine (A9785, Sigma-Aldrich, Inc.) |
|  | primocin (ant-pm-2, InvivoGen, San Diego, CA, USA) |
|  | estrogen (1 nM) (E2758, Sigma-Aldrich, Inc.) |
|  | retinoic acid (100 nM) (11017, Cayman Chemical, Ann Arbor, MI, USA) |
|  | triiodothyronine (0.2 pg/ml) (T5516, Sigma-Aldrich, Inc.) |
| Antibodies |  |
| Immunohistochemistry |  |
|  | E-cad (Clone 36, Ventana Medical Systems, Tucson, AZ, USA) |
|  | vimentin (Clone V9, Ventana Medical Systems, Tucson, AZ, USA) |
|  | p53 (Clone DO-7, Ventana Medical Systems) |
| Immunofluorescence staining |  |
|  | E-cad (ab1416, Abcam, Cambridge, UK) |
|  | Vimentin (GTX629744 and 100619, GeneTex, Irvine, CA, USA) |
|  | ZEB1 (HPA027524, Sigma-Aldrich, Inc.) |
|  | ZEB2 (HPA003456, Sigma-Aldrich, Inc.) |
|  | CREB3L1 (HPA024069, Sigma-Aldrich, Inc.) |
|  | Alexa Fluor 594 goat-anti-rabbit IgG (Invitrogen, Carlsbad, CA, USA) |
|  | Alexa Fluor 594 goat-anti-mouse IgG (Invitrogen) |
|  | Alexa Fluor 488 goat-anti-rabbit IgG (Invitrogen) |
|  | Alexa Fluor 488 goat-anti-mouse IgG (Invitrogen) |
| Western blotting |  |
|  | E-cad (RM-2100-S, Thermo Fisher, Waltham, MA, USA) |
|  | Vimentin (GTX100619, GeneTex) |
|  | ZEB1 (HPA027524, Sigma-Aldrich, Inc.) |
|  | ZEB2 (HPA003456, Sigma-Aldrich, Inc.) |
|  | CREB3L1 (HPA24069, Sigma-Aldrich, Inc.) |
|  | N-cadherin (sc-7939, Santa Cruz Biotech.) |
| Flow cytometry |  |
|  | Alexa Fluor 488 anti-human E-Cadherin (324110, BioLegend) |
|  | PE anti-human vimentin (IC2105P, R&D, Minneapolis, MN, USA) |
| RNA profiling in situ hybridization |  |
|  | Syto 13 (Nanostring Technologies, Seattle, WA, USA) |
|  | PanCK (NBP2-33200AF647, Novus Biologicals, Centennial, CO, USA) |
|  | Vimentin (sc-373717, Santa Cruz Biotech.) |
