# Supplementary material for: Characteristics and transcriptional regulators of spontaneous epithelial–mesenchymal transition in genetically unperturbed patient-derived non-spindled breast carcinoma
Source: Breast Cancer Res. 2024 Sep 10;26:130. doi: 10.1186/s13058-024-01888-5 (PMC11385830; doi:10.1186/s13058-024-01888-5)
Supplement: Supplementary file 17 — Supplementary Material 17: Supplementary Table S2 Primer sequences used in qRT-PCR [file 13058_2024_1888_MOESM17_ESM.docx]

**Supplementary Table S2** Primer sequences used in the qRT-PCR

| Gene | Forward sequence | Reverse sequence |
| --- | --- | --- |
| *ZEB1* | CCTGTCCATATTGTGATAGAGGC | ACCCAGACTGCGTCACATGT |
| *ZEB2* | AATGCACAGAGTGTGGCAAGGC | CTGCTGATGTGCGAACTGTAGG |
| *CREB3L1* | GCCTTGTGCTTTGTTCTGGTGC | CCGTCATCGTAGAATAGGAGGC |
| *CDH1* | AATTCCTGCCATTCTGGGGA | AATTCCTGCCATTCTGGGGA |
| *Vim* | AGCTAACCAACGACAAAGCC | TCCACTTTGCGTTCAAGGTC |
| *ID1* | GTTGGAGCTGAACTCGGAATCC | ACACAAGATGCGATCGTCCGCA |
| *ID3* | CAGCTTAGCCAGGTGGAAATCC | GTCGTTGGAGATGACAAGTTCCG |
| *MXD4* | GGAGCATCGTTTCCTGAAGCGG | GCCAAACTCCATGCCCTCTATG |
| *FOXO1* | CTACGAGTGGATGGTCAAGAGC | CCAGTTCCTTCATTCTGCACACG |
| *SMARCA1* | AACCTGGCAAGTGCTGATGTGG | CAGTGTTGTCAGTGATGAGACGG |
| *ZBTB16* | GAGCTTCCTGATAACGAGGCTG | AGCCGCAAACTATCCAGGAACC |
| *HIPK2* | AGCGTCATCACCATCAGCAGTG | AGTCGTGGACTGTGACACAGCT |
| *GAPDH* | AGCCTCAAGATCATCAGCAATGCC | TGTGGTCATGAGTCCTTCCACGAT |
