# Supplementary material for: Characteristics and transcriptional regulators of spontaneous epithelial–mesenchymal transition in genetically unperturbed patient-derived non-spindled breast carcinoma
Source: Breast Cancer Res. 2024 Sep 10;26:130. doi: 10.1186/s13058-024-01888-5 (PMC11385830; doi:10.1186/s13058-024-01888-5)
Supplement: Supplementary file 18 — Supplementary Material 18: Supplementary Table S3 RNA-seq results (TPM) for top enriched TFs in the M (HM, M-sc1, and M-sc5) and E (HE, E-sc60, and E-sc66) groups [file 13058_2024_1888_MOESM18_ESM.docx]

**Supplementary Table S3** RNA-seq results (TPM) of top enriched TFs in the M (HM, M-sc1, and M-sc5) and E (HE, E-sc60, and E-sc66) groups.

| **Gene** | **HE** | **E-sc60** | **E-sc66** | **HM** | **M-sc1** | **M-sc5** | **Mean HE group** | **Mean HM group** | **Fold (HM/HE group)** |
| --- | --- | --- | --- | --- | --- | --- | --- | --- | --- |
| *HLX* | 0.28 | 0.17 | 0.05 | 35.13 | 7.98 | 7.55 | 0.17 | 16.89 | 101.32 |
| *CREB3L1* | 0.43 | 0.36 | 0.27 | 53.17 | 15.67 | 8.18 | 0.35 | 25.67 | 72.66 |
| *ZEB2* | 0.14 | 0.07 | 0.16 | 6.05 | 1.78 | 1.10 | 0.12 | 2.98 | 24.14 |
| *EN1* | 0.44 | 0.10 | 0.11 | 9.24 | 4.01 | 2.40 | 0.22 | 5.22 | 24.08 |
| *HR* | 7.25 | 2.76 | 1.79 | 74.03 | 23.22 | 37.09 | 3.93 | 44.78 | 11.38 |
| *ZEB1* | 0.80 | 0.11 | 1.06 | 15.13 | 3.95 | 2.98 | 0.66 | 7.35 | 11.20 |
| *MAFB* | 3.03 | 0.87 | 0.62 | 26.22 | 3.85 | 1.97 | 1.51 | 10.68 | 7.09 |
