# Supplementary material for: Characteristics and transcriptional regulators of spontaneous epithelial–mesenchymal transition in genetically unperturbed patient-derived non-spindled breast carcinoma
Source: Breast Cancer Res. 2024 Sep 10;26:130. doi: 10.1186/s13058-024-01888-5 (PMC11385830; doi:10.1186/s13058-024-01888-5)
Supplement: Supplementary file 19 — Supplementary Material 19: Supplementary Table S4 Rank correlation between abundance of vimentin expression and 50 Hallmark signaling pathways [file 13058_2024_1888_MOESM19_ESM.docx]

| **Supplementary Table S4** Rank correlation between the abundance of vimentin expression and 50 Hallmark signaling pathways | | |
| --- | --- | --- |
|  | spearman's r | *p* value |
| EPITHELIAL_MESENCHYMAL_TRANSITION | 1 | 2.2251E-308 |
| INFLAMMATORY_RESPONSE | 1 | 2.2251E-308 |
| ALLOGRAFT_REJECTION | 1 | 2.2251E-308 |
| HYPOXIA | 1 | 2.2251E-308 |
| COMPLEMENT | 1 | 2.2251E-308 |
| INTERFERON_GAMMA_RESPONSE | 1 | 2.2251E-308 |
| UNFOLDED_PROTEIN_RESPONSE | 0.942857143 | 0.004804665 |
| APOPTOSIS | 0.885714286 | 0.018845481 |
| ANGIOGENESIS | 0.828571429 | 0.041562682 |
| MYOGENESIS | 0.828571429 | 0.041562682 |
| TNFA_SIGNALING_VIA_NFKB | 0.828571429 | 0.041562682 |
| XENOBIOTIC_METABOLISM | 0.828571429 | 0.041562682 |
| BILE_ACID_METABOLISM | 0.771428571 | 0.072396501 |
| COAGULATION | 0.771428571 | 0.072396501 |
| HEME_METABOLISM | 0.771428571 | 0.072396501 |
| IL6_JAK_STAT3_SIGNALING | 0.771428571 | 0.072396501 |
| INTERFERON_ALPHA_RESPONSE | 0.771428571 | 0.072396501 |
| PANCREAS_BETA_CELLS | 0.771428571 | 0.072396501 |
| MTORC1_SIGNALING | 0.657142857 | 0.156174927 |
| GLYCOLYSIS | 0.6 | 0.208 |
| PROTEIN_SECRETION | 0.6 | 0.208 |
| ADIPOGENESIS | 0.542857143 | 0.265702624 |
| IL2_STAT5_SIGNALING | 0.485714286 | 0.328723032 |
| UV_RESPONSE_DN | 0.485714286 | 0.328723032 |
| HEDGEHOG_SIGNALING | 0.428571429 | 0.396501458 |
| TGF_BETA_SIGNALING | 0.428571429 | 0.396501458 |
| WNT_BETA_CATENIN_SIGNALING | 0.428571429 | 0.396501458 |
| NOTCH_SIGNALING | 0.257142857 | 0.622787172 |
| REACTIVE_OXYGEN_SPECIES_PATHWAY | 0.2 | 0.704 |
| UV_RESPONSE_UP | -0.028571429 | 0.957154519 |
| KRAS_SIGNALING_UP | -0.085714286 | 0.87174344 |
| SPERMATOGENESIS | -0.085714286 | 0.87174344 |
| E2F_TARGETS | -0.2 | 0.704 |
| G2M_CHECKPOINT | -0.2 | 0.704 |
| PI3K_AKT_MTOR_SIGNALING | -0.2 | 0.704 |
| DNA_REPAIR | -0.257142857 | 0.622787172 |
| MITOTIC_SPINDLE | -0.314285714 | 0.544093294 |
| APICAL_JUNCTION | -0.371428571 | 0.468478134 |
| FATTY_ACID_METABOLISM | -0.428571429 | 0.396501458 |
| APICAL_SURFACE | -0.485714286 | 0.328723032 |
| MYC_TARGETS_V1 | -0.485714286 | 0.328723032 |
| OXIDATIVE_PHOSPHORYLATION | -0.485714286 | 0.328723032 |
| PEROXISOME | -0.485714286 | 0.328723032 |
| ANDROGEN_RESPONSE | -0.542857143 | 0.265702624 |
| P53_PATHWAY | -0.714285714 | 0.110787172 |
| MYC_TARGETS_V2 | -0.828571429 | 0.041562682 |
| CHOLESTEROL_HOMEOSTASIS | -0.942857143 | 0.004804665 |
| KRAS_SIGNALING_DN | -1 | 2.2251E-308 |
| ESTROGEN_RESPONSE_LATE | -1 | 2.2251E-308 |
