# Supplementary material for: Characteristics and transcriptional regulators of spontaneous epithelial–mesenchymal transition in genetically unperturbed patient-derived non-spindled breast carcinoma
Source: Breast Cancer Res. 2024 Sep 10;26:130. doi: 10.1186/s13058-024-01888-5 (PMC11385830; doi:10.1186/s13058-024-01888-5)
Supplement: Supplementary file 20 — Supplementary Material 20: Supplementary Table S5 Differentially significantly upregulated TFs (p < 0.05, log2FC ≥ 1) between each VIM subgroup versus VIM 0–1 subgroup [file 13058_2024_1888_MOESM20_ESM.docx]

**Supplementary Table S5** Differentially significantly upregulated TFs (*p* < 0.05, log2FC ≧1) between each VIM subgroup versus VIM 0-1 subgroup

| VIM subgroup | Transcription factor gene | *p* value | Log2FC |
| --- | --- | --- | --- |
| VIM 2-3 | *ZEB1* | 2.23E-11 | 1.53194 |
| VIM 3-4 | *ZEB1* | 8.6E-105 | 3.051455 |
|  | *ZEB2* | 5.57E-59 | 2.770664 |
|  | *SMARCA1* | 2.1E-29 | 1.851145 |
|  | *MXD4* | 6.28E-53 | 1.777336 |
|  | *ID3* | 7.24E-71 | 1.624571 |
|  | *ID1* | 3.38E-77 | 1.503987 |
|  | *ZBTB16* | 2.55E-16 | 1.354342 |
|  | *FOXO1* | 6.34E-15 | 1.085612 |
|  | *HIPK2* | 3.02E-19 | 1.010244 |
| VIM 4-5 | *ZEB1* | 3.3E-253 | 3.874247 |
|  | *ZEB2* | 4.7E-162 | 3.547321 |
|  | *SMARCA1* | 5.5E-135 | 2.768473 |
|  | *ID3* | 1.1E-130 | 2.071103 |
|  | *ZBTB16* | 5.66E-55 | 1.980038 |
|  | *GLIS3* | 5.66E-41 | 1.962894 |
|  | *ID1* | 1.2E-119 | 1.884263 |
|  | *MXD4* | 5.2E-84 | 1.860534 |
|  | *HIPK2* | 3.95E-89 | 1.709189 |
|  | *FOXO1* | 1.3E-61 | 1.687987 |
|  | *ETV1* | 1.1E-25 | 1.650542 |
|  | *TSC22D3* | 7.4E-21 | 1.388392 |
|  | *KLF7* | 1.68E-22 | 1.207834 |
|  | *TFPT* | 1.39E-68 | 1.13107 |
|  | *HIF1A* | 1.37E-88 | 1.117006 |
|  | *DEAF1* | 4.32E-37 | 1.028796 |
| VIM 5-6 | *CREB3L1* | 1.1E-101 | 8.218531 |
|  | *ZEB1* | 9.02E-47 | 3.779521 |
|  | *ZEB2* | 4.93E-48 | 3.54868 |
|  | *SMARCA1* | 3.93E-30 | 2.980248 |
|  | *ID3* | 8.95E-20 | 2.607282 |
|  | *GLIS3* | 1.57E-10 | 2.252223 |
|  | *ZBTB16* | 8.81E-17 | 2.082053 |
|  | *HIPK2* | 3.07E-21 | 2.010217 |
|  | *ID1* | 6.32E-16 | 1.940888 |
|  | *SNAI2* | 1.79E-06 | 1.935443 |
|  | *MXD4* | 1.11E-10 | 1.626905 |
|  | *TSC22D3* | 7.52E-09 | 1.605884 |
|  | *FOXO1* | 9.89E-07 | 1.528533 |
|  | *TFPT* | 4.67E-13 | 1.287562 |
|  | *HIF1A* | 5.11E-14 | 1.242291 |
|  | *CITED2* | 0.000136 | 1.162133 |
|  | *DEAF1* | 5.55E-05 | 1.081429 |
|  | *TWIST1* | 5.79E-07 | 1.014594 |
|  | *ZNF300* | 0.000907 | 1.013145 |
|  | *CREB3* | 8.34E-06 | 1.004661 |
|  | *NFKBIA* | 5.22E-07 | 1.004504 |
